# Greener synthesis of silver nanoparticles from *Zingiber officinale* rhizome extract for multidrug-resistant pathogen control, seed vigor enhancement, and fish embryo toxicity assessment

**DOI:** 10.3389/fcimb.2026.1798925

**Published:** 2026-05-08

**Authors:** Gajanan Sampatrao Ghodake, Min Kim, Jung-Suk Sung, Karthikeyan Chandrasekaran, Sandip Patil, Vini Mehta, Asad Syed, Ali H. Bahkali, Dae-Young Kim

**Affiliations:** 1 Department of Convergent Environmental Science, Dongguk University-Seoul, Goyang-si, Gyeonggi-do, Republic of Korea; 2 Department of Life Science, Dongguk University-Seoul, Goyang-si, Gyeonggi-do, Republic of Korea; 3 Department of Hematology and Oncology, Shenzhen Children’s Hospital, Shenzhen, China; 4 Paediatric Research Institute, Shenzhen Children’s Hospital, Shenzhen, China; 5 Global Research Cell, Dr. D. Y. Patil Dental College & Hospital, Dr. D. Y. Patil Vidyapeeth (Deemed to be University), Pimpri, Pune, India; 6 Department of Botany and Microbiology, College of Science, King Saud University, Riyadh, Saudi Arabia

**Keywords:** antibacterial activity, green synthesis, rhizome extract, seed priming, silver nanoparticles

## Abstract

**Background:**

Antibiotics are prone to multidrug resistance (MDR), underscoring the urgent need for safer, sustainable, and efficient antibacterial strategies. In this context, this account presents the green synthesis of silver nanoparticles (AgNPs) for multifunctional applications, including antimicrobial efficacy, agricultural seed priming, and ecotoxicological assessment using a zebrafish embryo model.

**Methods:**

A green chemistry–based synthesis of AgNPs is presented using *Zingiber officinale* extract as a biogenic reducing agent. The synthesized AgNPs were evaluated for antibacterial activity against MDR bacterial pathogens. Their potential benefits in agriculture were assessed through seed priming assays. Furthermore, the safety and toxicological effects of AgNPs were investigated using an *in vivo* zebrafish embryo model.

**Results:**

The AgNPs were characterized using XRD, HR-TEM, SAED, XPS, and FTIR, confirming a narrow nanoscale size distribution ranging from 4 to 14 nm, high crystallinity, and stable surface capping. The antibacterial activity results showed that the lethal concentration at 80% (LD_80_) of the synthesized AgNPs is approximately 50 ppm against both Gram-positive and Gram-negative bacterial pathogens. Seed priming assay results showed enhanced seed germination rate and vegetative growth across all tested AgNP concentrations (20 to 60 ppm). However, the results also showed that lower concentration ranges can be toxic to zebrafish embryos.

**Discussion:**

The research demonstrates the antibacterial effectiveness of the synthesized AgNPs using the rhizome extract. Moreover, AgNP-mediated seed priming can enhance seed vigor and vegetative growth in two important crops, *Trigonella foenum-graecum* and *Vigna radiata*. However, zebrafish embryo toxicity tests showed serious ecotoxicological concerns. It is proposed to use AgNPs at safe, effective concentrations, considering the risk–benefit to avoid adverse effects on the environment, human health, and aquatic life.

## 1 Introduction

The control of bacterial diseases is an important global public health issue, driven by the gradual decline in the efficacy of traditional antibiotic treatments (Breijyeh et al., 2020; Murray et al., 2022; Serwecińska, 2020; Wesseling and Martin, 2022). Antimicrobial resistance (AMR), including multidrug resistance (MDR), is widely recognized as a major and emerging global public health concern (Prestinaci et al., 2015). It compromises the efficacy of conventional antibiotic treatments, leading to prolonged illness, higher mortality rates, and the re-emergence of life-threatening bacterial infections (Murray et al., 2022; Walsh et al., 2023). Given that a post-antibiotic era is expected, in which current antibiotics may be less effective, the scientific community needs to increasingly focus on safer nanotechnology. In the present work, *Z. officinale* rhizome extract has been used as a green-chemistry-based reducing and stabilizing agent for the high-throughput synthesis of colloidal, stable suspensions of AgNPs.

Numerous nanomaterials (NMs) having potent antibacterial properties, including silver (Ag), zinc oxide (ZnO), titanium dioxide (TiO_2_), copper oxide (CuO), and a series of nanocomposites and bimetallic nanoalloys, are being considered as promising alternative antimicrobial agents (Ahmed et al., 2020; Chakraborty et al., 2022; Huang et al., 2023; Ibarra-Cervantes et al., 2024; Makabenta et al., 2021; Wang et al., 2024). Among the wide range of nanomaterials (NMs), the antibacterial properties of AgNPs exhibit intrinsic and potent antibacterial properties and warrant further exploration for promising applications in medicine (Acharya et al., 2020; Mahakham et al., 2017) and agriculture (Nile et al., 2022). However, the novelty of the studies lies in the integrated, multi-dimensional approach to the biosynthesized AgNPs, which targets their applications across the fields, including in medicine (Laib et al., 2025), nanosensors (Al-aqbi et al., 2024; Alaziz et al., 2025), agriculture (Kim et al., 2024), and the environment (McGillicuddy et al., 2017). However, the increased use of metal NPs has raised serious concerns about their toxicity in aquatic environments, underscoring the need to assess their environmental impact (Bolan et al., 2024; Wang et al., 2024).

Various plants, parts, extracts, and approaches for the preparation of metal NPS, including AgNPs, have been widely reported in literature (Chan et al., 2024; Das et al., 2025; Hatipoğlu et al., 2023; Irshad et al., 2023; Narayanan et al., 2023; Taipe Huisa et al., 2024; Tarannum et al., 2019; Vanlalveni et al., 2021). This account presents the significance of green chemistry, the optimization of nanosynthesis parameters for the *Z. officinale* extract method, and the potential of AgNPs for antibacterial and agricultural applications. *Z. officinale* rhizome is a rich source of biomolecules, including polyphenols, terpenoids, gingerol, carbohydrates, polypeptides, amino acids, dietary fiber, saponin, vitamins, and other nutritional compounds, possessing functional groups, for example, –OH, –COOH, –NH_2_, and –SH (Ajanaku et al., 2022; Laelago Ersedo et al., 2023; Ooi et al., 2022). Bioactive compounds, such as amino acids, phenolics, and terpenoids, facilitate the reduction of Ag ions to zero-valent AgNPs and simultaneously stabilize them in aqueous media (Akullo et al., 2023; Eisa et al., 2019).

In this context, the present study proposes a facile, green, and sustainable approach for the extracellular synthesis of AgNPs using biologically active *Z. officinale* rhizome extract as a dual reducing and stabilizing agent. In particular, the present study makes a unique contribution to the existing literature by exploring four major aspects and integrating them. This research presents a comprehensive and cross-disciplinary approach to integrate: (i) preparation and physicochemical characterization of the AgNPs; (ii) antibacterial evaluation against MDR pathogens; (iii) the potential of AgNPs as seed priming agents; and (iv) ecotoxicology testing using a zebrafish embryo model. This integrated approach proposes plant-mediated synthesis of AgNPs and supports the safe and efficient design of multifunctional, application-oriented AgNPs for sustainable biomedical and agricultural applications. The present study also aims to broaden the scope of AgNPs by discussing the risk-benefit relationship. Further, we proposed correlating the antimicrobial potency/agricultural benefits with potential toxicity concerns, thereby feasibility of safe deployment.

## 2 Methods and materials

### 2.1 Chemicals and reagents

Chemicals and reagents required for the experiment, such as silver nitrate, Gram’s iodine solution, crystal violet reagent, poly-L-lysine, and glutaraldehyde, were obtained from Aldrich Chemicals. A 1 M sodium hydroxide (NaOH) solution in deionized (DI) water and a 50 M phosphate buffer solution at pH 7.2 were purchased from Bio-sensing Chemicals, Korea. For microbial culture growth, analytical-grade Luria-Bertani broth, nutrient broth, and agar powder were obtained from Becton Dickinson Chemicals. Fresh ginger rhizomes were collected from local vegetable markets in South Korea, and a freshly prepared extract was used to synthesize AgNPs. MDR Gram-positive *Staphylococcus aureus* [KCCM:11335] and Gram-negative *Escherichia coli* [KCCM:11234] were obtained from the Korean Culture-Center of Microorganisms, Korea. In this regard, double-distilled (DI) water (Thermo Scientific Apparatus) was used in this investigation to ensure appropriate chemical reactions without compromising the quality of the reaction yield.

### 2.2 Biosynthesis of AgNPs

A stock solution of aqueous rhizome extract was prepared at about 20% concentration using a blender in DI water. The rhizome extract was filtered through Whatman No. 1 filter paper, subjected to ultracentrifugation at 12,000 rpm for three 15-minute cycles, and then used to obtain a clear solution free of suspended particles. Biosynthesis was performed in a 10 mL reaction mixture at 35 °C, and different parameters were systematically optimized to assess their influence on biosynthesis and yield. AgNP formation was monitored over 12 hours under selected reaction conditions. The parameters tested include the NaOH concentration, the extract concentration, and the AgNO_3_ concentration. The objective of these parameters was to achieve the desired results for yield and stability of the colloidal suspensions. The NaOH concentration ranged from 0.5 to 5.0 mM, whereas the extract and AgNO_3_ concentrations were kept constant at 2.0% w/v and 0.4 mM, respectively. Furthermore, to study the kinetics and efficiency of AgNP fabrication, the AgNO_3_ concentration was varied from 0.25 to 4.0 mM. In comparison, the concentrations of NaOH (3 mM) and the extract were kept constant at 2.0% w/v. Lastly, the synthesis of AgNPs was conducted under optimized reaction conditions, including the extract concentration (2.0% w/v), NaOH (3 mM), and AgNO_3_ (0.4 mM), in a more environmentally friendly manner. The biosynthesized AgNPs, under optimized conditions, were then purified by ultracentrifugation at 14,000 rpm for 15 minutes in two cycles. Subsequently, the AgNPs were characterized in detail before being used to determine the antibacterial activity, seed priming, and ecotoxicological impacts.

### 2.3 Characterization of AgNPs

The investigation of the AgNP samples used multiple analytical tools. The UV-Vis absorbance spectra of the AgNP samples prepared at varying concentrations were first diluted in DI water, and the AgNPs were then analyzed. The high-resolution transmission electron microscopy (HR-TEM) technique has been employed to obtain the images of the AgNPs and their electron diffraction patterns. HR-TEM samples of the AgNP solution containing 15µL of the solution were prepared using the copper grid designed specifically for this purpose. The oxidation state and core-level spectrum of elements of the AgNPs were measured using X-ray photoelectron spectroscopy (XPS). The crystalline nature of the AgNPs was studied using a PANalytical X’Pert Pro XRD diffractometer from Malvern Panalytical, United Kingdom. Fourier transform infrared spectroscopy (FTIR) was used to examine the chemical properties of the biomolecules in the extract and AgNPs.

### 2.4 Bacterial cell culture

The present study evaluates the antibacterial potential of AgNPs against MDR bacteria, such as *Staphylococcus aureus* [KCCM: 11335] and *Escherichia coli* [KCCM: 11234]. Several microbiological assays were carried out, including cell viability assays, colony-forming unit (CFU) assays, the disk diffusion method, and morphological imaging techniques. MDR strains were cultured on nutrient-enriched agar plates under controlled conditions at 35 °C for 24 hours. Afterward, the bacteria were retrieved and suspended in nutrient broth to allow them to multiply and reach the stationary growth phase. Subsequently, the cultures were centrifuged at 7500 rpm for 7 minutes. This bacterial pellet was resuspended in sterile culture medium, and a fresh bacterial suspension was used as the inoculum, with the bacterial cell density maintained at 2 × 10^3^ CFU/mL. while AgNO_3_ was excluded as it reacts immediately with cellular structures and biomolecules.

#### 2.4.1 Colony count method

Further, this study aims to elucidate the antibacterial potential of AgNPs by assessing their ability to reduce the formation of viable bacterial colonies following treatment. The colony count assay was used to assess the antibacterial potential of AgNPs. The bacterial cultures in the stationary phase were treated with AgNPs at concentrations of 30–200 ppm for 4 hours at 37 °C. After treatment, 200 μL of each culture was plated onto an agar plate and incubated at 37 °C for 24 hours under stationary conditions. The number of visible bacterial colony-forming units (CFU) present on the surface of a nutrient agar Petri dish was used to estimate the viable bacterial population. For comparative purposes, negative control plates were also prepared by inoculating bacterial cells onto nutrient agar without AgNP treatment. The percentage reduction in CFUs was calculated using an equation previously established in the literature report (Shao et al., 2015). Relative CFU killing (%) = [1 ― Number of colonies on AgNP-treated plates/Number of colonies on control plates] × 100.

#### 2.4.2 Cell viability

The turbidity assay measured bacterial cell viability to assess the impact of AgNPs on bacterial strains, including *S. aureus* and *E. coli*. To assess the bactericidal activity of AgNPs, the colloidal solutions were added to sterile Luria-Bertani broth at concentrations ranging from 20 to 200 ppm. Fresh bacterial cultures were inoculated into samples containing different concentrations of AgNPs. The culture samples were incubated at 37 °C and 120 rpm on a shaker under standardized conditions. The density of the bacterial population was determined by measuring the absorbance at 600 nm using a Tecan ELISA reader (Männedorf, Switzerland). To analyze the collected data, one-way ANOVA was used to assess differences in AgNP concentration. Lethal dose (LD) values for AgNPs that significantly inhibited bacterial cells were established. To confirm the reliability of the results, the experiment was carried out using sterile culture media under the identical culture conditions as the control. The absence of turbidity in the bacterial culture after treatment with different concentrations of AgNPs indicated reduced bacterial cell viability.

#### 2.4.3 Imaging AgNPs-treated bacterial cells

Morphological changes in the bacterial cells after the application of AgNPs were studied using optical microscopy and field emission scanning electron microscopy (FE-SEM). Bacterial cultures in the early stationary phase were exposed to an AgNP solution at 50 ppm for 4 hours. The subsequent steps included Gram staining of the bacterial cells, following the procedure described in a previous report (Ghodake et al., 2020), to examine morphological changes after AgNP treatment. Optical micrographs of Gram-stained bacterial cells treated with AgNPs were obtained to study morphological modifications. Gram staining was used to study the surface morphology of AgNP-treated bacterial cells using optical microscopy. Sterile culture medium without antibacterial agents was used as the standard negative control. A sterile culture medium without AgNPs was used as the standard negative control.

To investigate nanoscale changes, field-emission scanning electron microscopy (FE-SEM) was used to examine the bacterial strains under study. The preparation of bacteria for FE-SEM examination is similar to early sample preparations used in Gram staining and begins with fixation in 2.5% glutaraldehyde. The fixed bacteria were placed on coverslips coated with poly-L-lysine. A gradual dehydration of the bacteria was achieved by treating them sequentially with ethanol-DI water solutions, increasing the ethanol content from 20% to 100%. Following this, the dried bacteria were coated with a thin layer of platinum to enable conductivity for FE-SEM imaging. The bacterial samples were examined at specific points on their surfaces using FE-SEM (Hitachi S-4700, Tokyo, Japan), providing images of bacterial morphological structures.

### 2.5 Seed priming studies

#### 2.5.1 Seeds selection

This study assessed the application of AgNPs in seed priming, germination rate, and growth of *Trigonella foenum-graecum* (fenugreek) and *Vigna radiata* (mung bean) under saline stress conditions. Dry and clean seeds were first washed with DI water, then treated with a 1% sodium hypochlorite solution for 5 minutes to eliminate surface-borne pathogens. After surface sterilization, seeds were washed again with sterile DI water to remove any remaining chemical residues. Subsequently, the seeds were divided into five groups, with 60 seeds per group to ensure uniformity in the experiments. Each group was assigned a label based on the concentration of the AgNP solution: 20, 30, 40, 50, or 60 ppm. The seeds were then exposed to the respective solutions for 4 hours to allow the AgNPs to adhere to their surfaces. Finally, primed seeds were then dried at room temperature using clean absorbent tissue.

#### 2.5.2 Seed germination assay

A 50 millimolar (mM) NaCl solution was freshly prepared in soil-treated DI water to simulate saline irrigation conditions and mimic salt stress in an agriculture environment. The petri dishes were then set up, labeled, and assigned to different seed types. Tissue towels covering the entire surface of each petri dish were uniformly moistened with 50 mM NaCl solutions. Dried pristine seeds, hydro-primed seeds, and AgNP-primed seeds were counted and evenly distributed over the wetted surface to avoid overcrowding. The Petri dishes were then sealed to ensure dark conditions and high humidity, and to prevent contamination. The seeds were germinated in the dark at an ambient temperature of 24± 2 °C. Seed germination was monitored by radicle emergence. The lengths of radicles and roots were recorded at 12-hour intervals from 24 to 36 hours, in centimeters. The appearance of plumules and radicles was taken as an indicator of seed germination. The appearance of plumules and radicles indicates the proportion of seeds from which radicles and roots developed. Photographs of the germinated seeds were taken at 36 hours. The experiment was carried out in triplicate across seven treatments. The treatments included two controls: unprimed seeds and hydro-primed seeds. The other five treatments included seeds primed with AgNP solutions, with all other conditions held constant.

#### 2.5.3 Seedling growth assay

Healthy, well-germinated seedlings were selected from the control group and the AgNP-primed groups. A compost mixture was prepared using garden mix compost, and healthy seedlings were transferred to the compost to avoid mechanical damage. The trays were kept under a 12/12-hour light/dark cycle at ambient temperature. The trays were frequently irrigated to maintain moisture levels. Light, temperature, and humidity were kept constant throughout the experiment. The lengths of the roots and stems were observed after five days of sowing the well-germinated seeds. The healthy seedlings were carefully removed from the compost to measure the lengths of their roots and stems without damaging them. The lengths of the roots and stems were recorded. A comparative analysis was conducted of the elongation of roots and stems in seedlings from the control and AgNP-primed groups. Statistical analysis was conducted to evaluate the priming effect of AgNP under ambient conditions, and the results were presented as histograms.

### 2.6 Zebrafish embryos model toxicity assay

#### 2.6.1 Maintenance of zebrafish and embryo collection

Adult *Danio rerio* (male and female, 4 months old) of the wild-type strain were maintained under standard laboratory conditions solely for the purpose of obtaining fertilized embryos. The fish were housed in a controlled breeding facility at 28.5 ± 0.5 °C under a 14/10 h light/dark cycle, with water maintained at pH 7.2 ± 0.3 and dissolved oxygen levels of approximately 6.0 ppm. On the evening before spawning, male and female fish were placed in breeding tanks at a 2:1 ratio, separated by a steel mesh grid to prevent egg predation. The fish were left undisturbed overnight, and the following morning, fertilized eggs were collected with Pasteur pipettes and examined under a stereomicroscope. Unfertilized or damaged embryos were discarded, and only healthy blastula-stage embryos were selected. These embryos (≤120 hpf) were subsequently used to assess acute toxicity to AgNPs.

#### 2.6.2 Collection and exposure of the embryos to nanoparticles

For toxicity testing, 10 healthy embryos were placed in the wells of a 24-well plate, along with 1 ml of embryo water. Two different nanoparticle concentrations, 10 ppm and 20 ppm, were added to the wells and incubated for 96 hours at 28.5 °C.

#### 2.6.3 Evaluation of survival rate, hatching rate, and gross morphological changes

Each of the ten embryos was placed on a 6-well plate containing 3 mL of embryo medium. The embryos were then gradually treated with AgNPs at effective concentrations of about 10 ppm and 20 ppm between 6 and 96 hours post-fertilization (hpf). At each time point (24, 48, 72, and 96 hpf), the survival rate, hatching time, and gross morphological changes were studied. The number of dead, non-hatched, or hatched embryos, as well as the number of malformed embryos, was discarded. Using a stereoscopic microscope (Magnus, India), the embryos were viewed under bright-field illumination, and photographs were obtained.

#### 2.6.4 Ethics statement

All experiments were conducted exclusively on zebrafish embryos within 120 hpf. At this developmental stage, zebrafish embryos are not classified as protected animals under current animal experimentation regulations. For zebrafish, ethical clearance is required 5 days post-fertilization. Therefore, formal ethical approval is not required for this study. Although, the work was performed outside Europe, all procedures were conducted in accordance with internationally recognized animal welfare principles and in alignment with Directive 2010/63/EU on the protection of animals used for scientific purposes. The zebrafish embryo toxicity experiments were carried out at the KIRND Institute of Research and Development Pvt. Ltd., India.

### 2.7 Statistical analysis

All experiments were performed in three independent biological replicates to ensure reproducibility and reliability of the results. Data obtained from these independent experiments were used for subsequent statistical analysis. One-way analysis of variance (ANOVA) was performed and the results are expressed as means ± standard error of the mean. Sigma plot and Origin were used for data visualization and curation.

## 3 Results and discussion

### 3.1 Biosynthesis of AgNPs

The purpose of this study is to identify a safe, cost-effective, and efficient route for the biosynthesis of AgNPs and to assess their potential for use in medicine, agriculture, and environmental science. In our study, the conditions of the AgNP synthesis route were optimized to minimize side-product formation and maximize AgNP formation. Thus, AgNP synthesis via the extract route, after temperature studies, found that ambient conditions of 35 °C are the most suitable alternative to energy-intensive methods. In our first trial of AgNP synthesis via the extract route, no color change was observed during the reaction of the extract with AgNO_3_. Initiation of AgNP formation was observed immediately upon addition of a 3 mM NaOH solution to the AgNO_3_ solution, consistent with previous findings (Yadav et al., 2017). The rapid appearance of a dark yellow color in the solution at ambient temperature is consistent with green chemistry (Duan et al., 2015).

Initially, our research focused on the reaction time frame during which AgNPs form during the reduction of the extract. Later, it was used to adjust reaction time when measuring the results of the latter parts of the research. To acquire a comprehensive understanding of the phenomenon, the UV-Vis spectra of the resulting AgNP solution were recorded over the course of the reaction, spanning 20 to 720 minutes. The real-time appearance of the surface plasmon resonance band in the reaction mixture was observed in the UV-Vis spectrum, with a consistent increase, especially the band centered at 420 nm, which corresponds to the plasmon wavelength (λmax) (**Supplementary Figure 1A**). Although the reduction of AgNO_3_ to AgNPs occurs rapidly, with unique UV-Vis spectra, within only 20 minutes of the reaction time frame of the synthesis of AgNPs. The results of the experiment revealed that a sufficient time was required to initiate the reduction of AgNO_3_, as evidenced by characteristic UV-Vis spectra of AgNP formation. The linear increase in yield indicates controlled growth of the AgNPs and further suggests the extract’s strong reducing potential. The absorbance at 420 nm increased linearly and then saturated over 360 minutes of the reaction, remaining stable with further incubation, suggesting stability of the colloidal solutions (**Supplementary Figure 1B**). This further reveals that the method is environmentally friendly, suitable for synthesizing NPs, and capable of producing monodisperse, particularly stable AgNPs via the classical nucleation and growth mechanism (Polte, 2015). The formation of AgNPs was monitored over 12 hours of incubation under the selected reaction conditions, and the remaining parameters were systematically investigated to optimize the green synthesis method.

**Figure 1:**
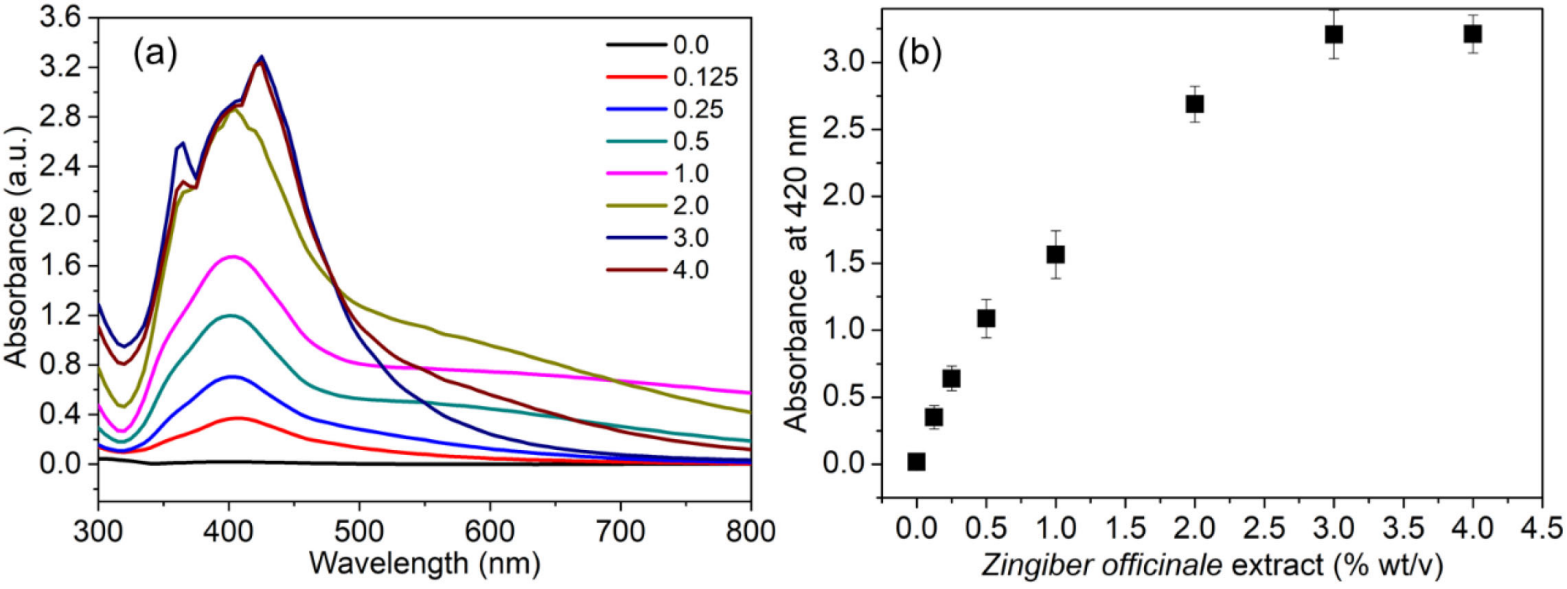
Influence of *Z. officinale* rhizome extract on the green synthesis of AgNPs. **(A)** UV–visible absorption spectra illustrating the formation of AgNPs at varying concentrations of *Z. officinale* rhizome filtrate, characterized by the surface plasmon resonance (SPR) peak around 420 nm. **(B)** The corresponding absorbance intensity at 420 nm indicates the relative yield of AgNPs as the extract concentration increases.

**Figure 1A** shows the UV-Vis spectra for the AgNPs obtained from the samples containing different extract ratios. From the UV-Vis spectra, it is clear that the samples contained mixtures of AgNPs, as evidenced by a broad plasmon bandwidth and an SPR peak at approximately 420 nm. An increase in the extract solution concentration from 0.125 to 4.0% (wt/v) was accompanied by an increase in the absorption peak. When the extract concentration was between 0.5 and 2.0% (wt/v), the absorption peak was at 420 nm, with a broad plasmon bandwidth. An increase in the absorption peak was observed at an extract solution ratio of 3.0% (wt/v), the maximum among the samples, compared with the absorption peak observed for the sample obtained at 0.125% (wt/v) solution. The UV-Vis spectrum corresponding to the desired sample of AgNPs was observed at 3.0% (wt/v) solution. A linear increase in the absorbance intensity was observed simultaneously with the SPR peak throughout the range of the extract solution concentrations from 0.125 to 3.0% (w/v), peak corresponding to the maximum absorbance intensity or yield of AgNPs (**Figure 1B**). Similar results have also been observed elsewhere; the change in plasmon bandwidth indicates the narrow size distribution of the obtained AgNPs, which the careful regulation of the reducing agent concentrations can control. Although the green chemistry principle of atom economy is primarily used in chemical synthesis, it also needs to be fully applied to the synthesis of NPs (Castiello et al., 2023; Duan et al., 2015).

**Figure 2:**
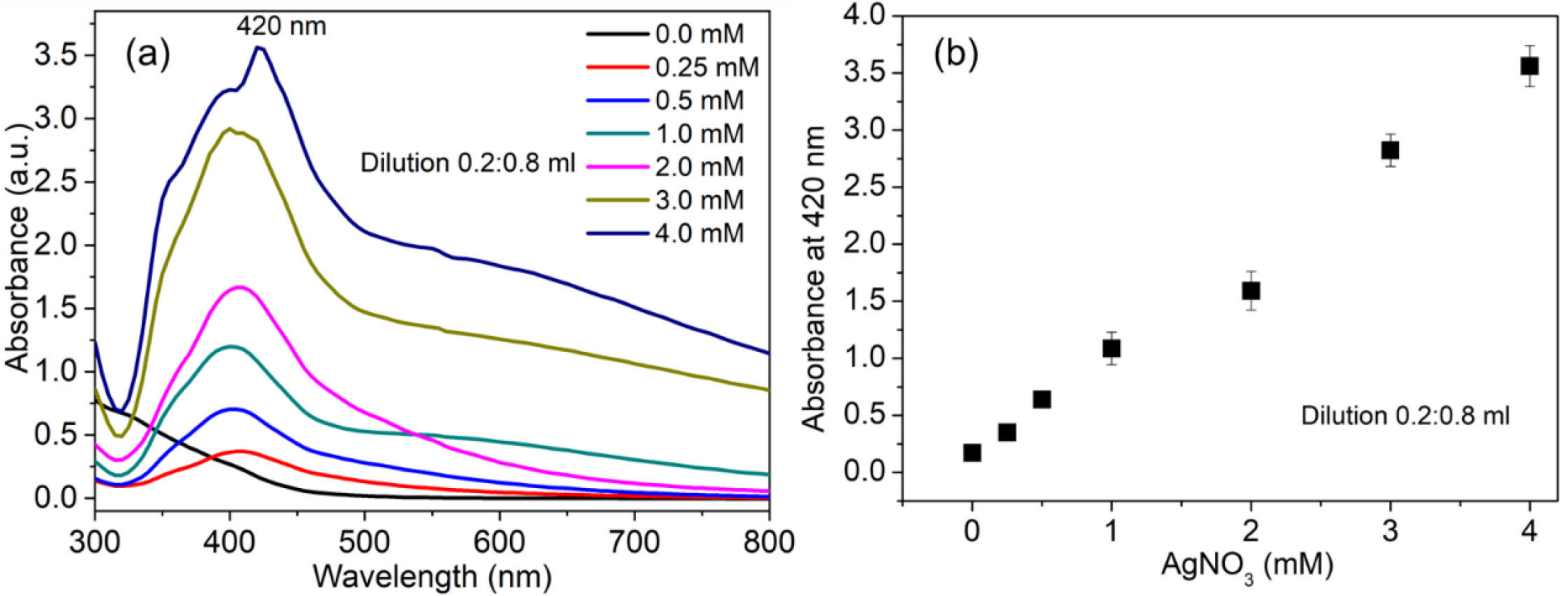
Demonstration of the efficacy of the *Z. officinale* rhizome filtrate–mediated synthesis method. **(A)** UV–visible absorption spectra showing the formation of AgNPs with increasing concentrations of the metal precursor AgNO_3_. **(B)** Corresponding absorbance intensity at 420 nm indicating the relative yield of AgNPs at different AgNO_3_ concentrations.

This study further investigated the kinetics of AgNP synthesis by varying the AgNO3 concentration while keeping a constant extract/NaOH ratio. **Figure 2** shows the UV-Vis spectra and the absorbance parameters of the AgNPs at different AgNO_3_ concentrations. The SPR band of the AgNPs remained centered at 420 nm. Still, the SPR band width continued to widen as the AgNO_3_ concentration increased from 0.25 to 4.0 mM (**Figure 2A**). Thus, the potential of the extract in converting a higher range of AgNO_3_ concentrations suggests the possibility of enhancing the yield of the AgNPs. One of the observed phenomena was the linear increase of the SPR band intensity of the AgNPs as the AgNO_3_ concentration increased (**Figure 2B**). It has been established that the concentration of the AgNPs is proportional to the extinction coefficient of the SPR band of AgNPs (Alzoubi et al., 2023). There was, however, no deviation from the linear relationship of the SPR band intensity of the AgNPs as a function of AgNO_3_ concentration at all tested concentrations, as seen in **Figure 2B**, indicating that this method is highly efficient in obtaining a higher yield of AgNPs (Quintero-Quiroz et al., 2019). This report showcases the potential of green synthesis methodologies for scaling up synthesis when yield is crucial and for developing various applications (Studart et al., 2007).

**Figure 3:**
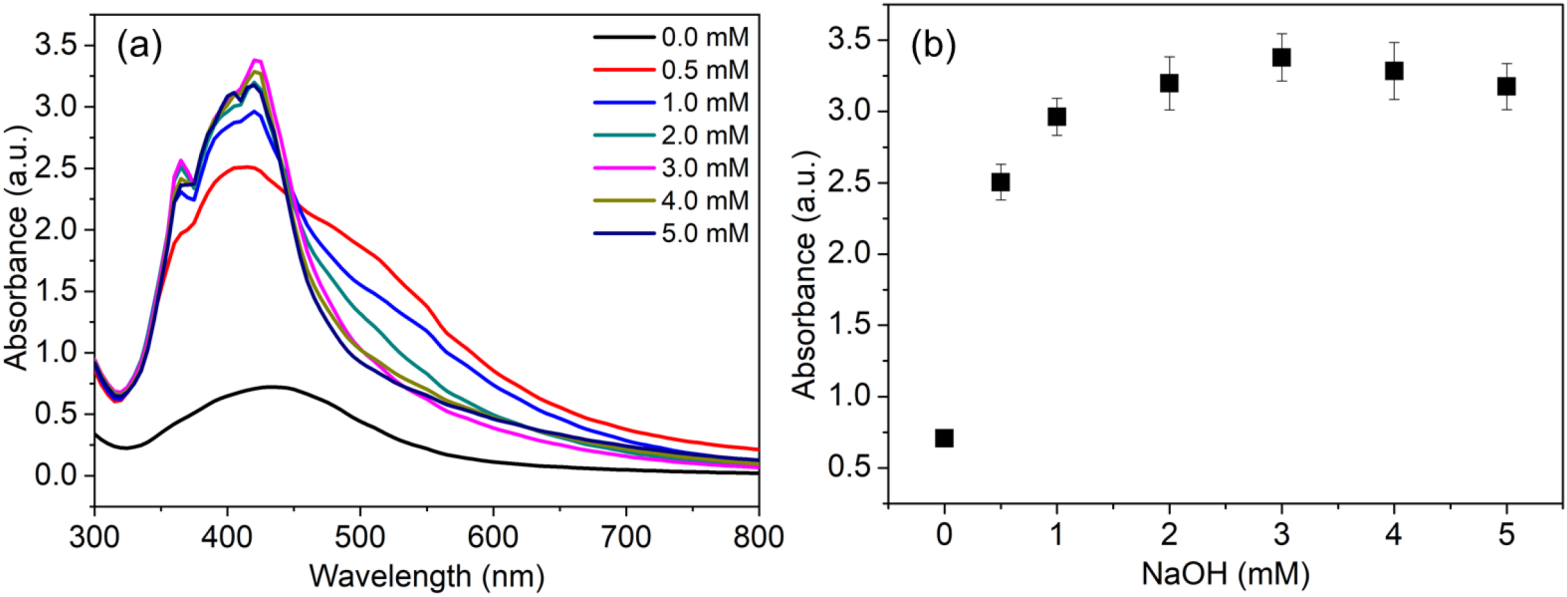
Effect of alkaline pH on the green synthesis and yield of AgNPs. **(A)** UV–visible absorption spectra illustrating the emergence and intensification of the SPR band as NaOH concentration increases. **(B)** Corresponding absorbance intensity at 420 nm used to estimate the relative yield of AgNPs.

The influence of NaOH on the reduction rate of Ag^+^ ions to Ag^0^ and on the formation of AgNPs provides insight into the mechanism of synthesis. Ag^+^ ions are immediately reduced to Ag0, as evidenced by a rapid color change to light yellow, indicating a significant role for alkaline conditions (Kim et al., 2017). The impact of alkali was examined by varying the NaOH concentration from 0.5 to 5 mM at 35 °C, during which the UV-Vis absorption spectra exhibited a single SPR peak across all samples (**Figure 3A**), indicating that the yield of AgNPs is directly proportional to the NaOH concentration. The red shift in the UV-Vis spectra towards longer wavelengths in samples containing low concentrations of NaOH (0.5–2.0 mM) indicates larger particles and low yield (Darroudi et al., 2010).

The relationship between absorbance intensity without and with concentration of NaOH has shown a significant difference in the production of AgNPs, a sharp SPR peak was evident for minor concentration of NaOH (0.5 mM) (**Figure 3B**). Among the molar ratios studied, it has been found that 3 mM concentration of NaOH is optimal for the synthesis process, which corresponds to maximum production with desired physicochemical properties of AgNPs. This phenomenon persists up to 4 mM NaOH (**Figure 3A**). The production of AgNPs, as measured by the absorbance at 420 nm, remains at its maximum plateau with further increases in NaOH concentration. This close relationship between the concentration of NaOH and the controlled production of AgNPs suggests that a concentration of NaOH is a critical factor in optimal production of AgNPs. From a scientific perspective, as reported previously, alkaline conditions promote deprotonation of functional groups, thereby facilitating the reduction reaction (Kim et al., 2018) and improving the surface stability of AgNPs (Panigrahi et al., 2006). The positive influence of dilute NaOH concentrations, as observed in the present method, underscores how readily the classical nucleation process and subsequent isotropic growth can be fine-tuned (Whitehead et al., 2021).

**Figure 4:**
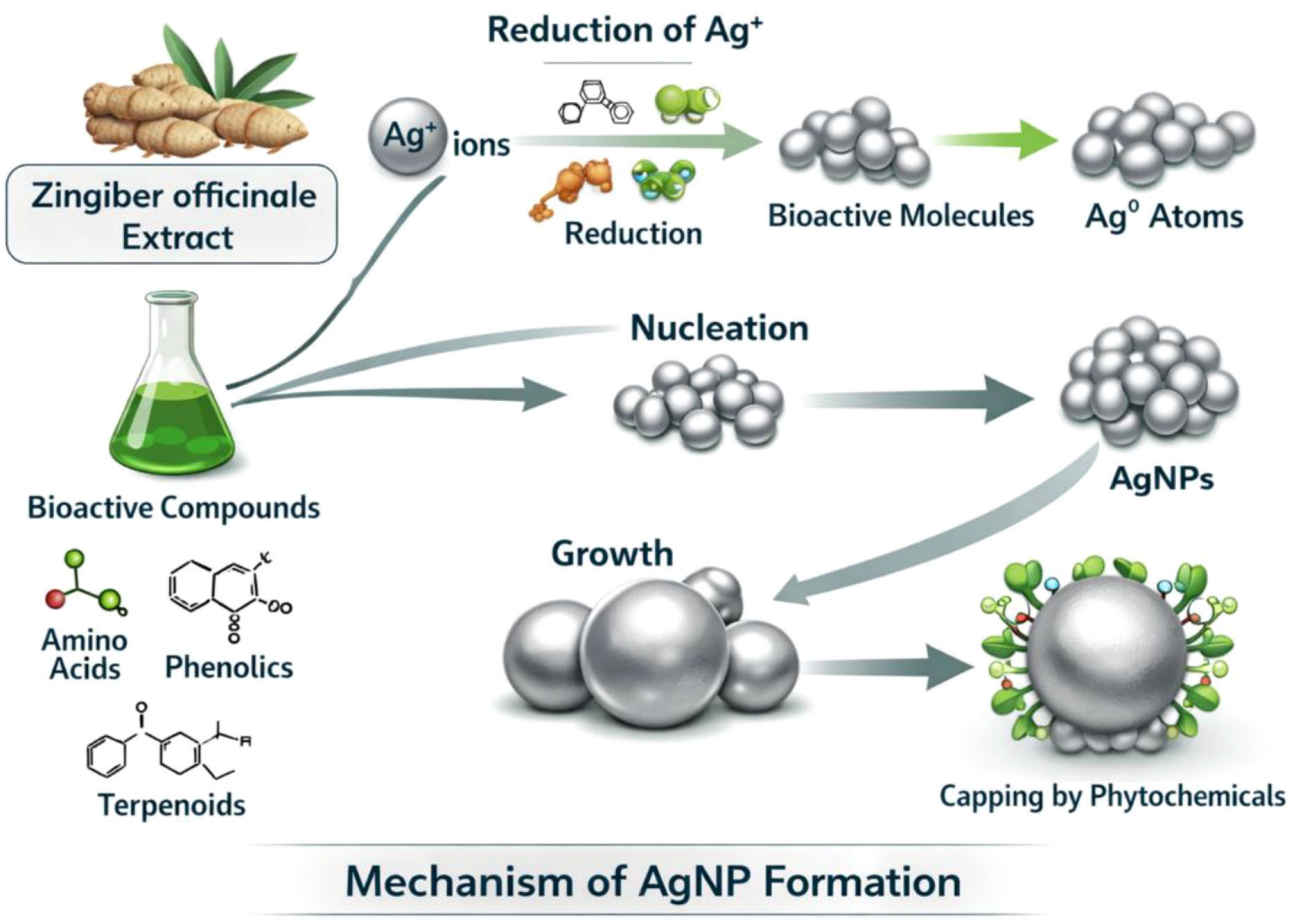
A schematic figure illustrating the mechanism of AgNP formation, showing the reduction of Ag^+^ ions by phytochemicals in *the Z. officinale* extract, followed by nucleation, growth, and stabilization.

Further explore the physicochemical mechanisms underlying the reduction, nucleation, growth, and stabilization of AgNPs in the *Z. officinale* rhizome extract, with particular attention to bioactive phytoconstituents. The numerous bioactive compounds, such as phenolic compounds, flavonoids, terpenoids, amino acids, and sugars (Mao et al., 2019), present in the rhizome extract of *Z. officinale* can play an important role in the biosynthesis of AgNPs. Typically, Ag^+^ ions readily react with phytochemicals in the extract, especially phenolic compounds and flavonoids with hydroxyl (α OH) groups, which donate electrons to facilitate the reduction of the Ag^+^ ions to Ag^0^ metal (Fahim et al., 2024). Likewise, terpenoids and other reducing biomolecules participate in redox reactions that facilitate the synthesis of Ag atoms. After this reduction step, the Ag^0^ atoms that form undergo nucleation, forming small clusters or nuclei, and reaction parameters govern the number of AgNPs (Lee and Jun, 2019). Subsequently, as stable nuclei get formed, Ag crystals start to build on top of these nuclei to form mature AgNPs as illustrated in **Figure 4**. The overall mechanism and tested parameters suggest that the proposed method is appropriate for controlling the overall yield, size distribution, and shapes of AgNPs, using factors such as pH, reaction time, precursor and extract concentrations, etc.

This green synthesis fully complies with the principles of green chemistry. In the context of green chemistry, the process of synthesizing AgNPs, as presented in the given method, uses readily available reagents such as AgNO_3_ and dilute NaOH solutions, no other manmade hazardous chemicals. A readily available, safe, natural *Z. officinale* extract serves as an alternative, milder approach to the bottom-up biosynthesis of the desired AgNPs by efficiently reducing metal precursors as reported previously (de Oliveira et al., 2020). The green process is presented for synthesizing AgNPs abides green chemistry principles such as atom economy (P2) and real-time monitoring (P11), the process can be made more efficient, prevents pollution (Feder-Kubis et al., 2024), minimizes byproducts formation and reduces overall environmental impact, ensuring the process’s sustainability (kazemi et al., 2023).

### 3.2 Characterization of AgNPs

**Figure 5:**
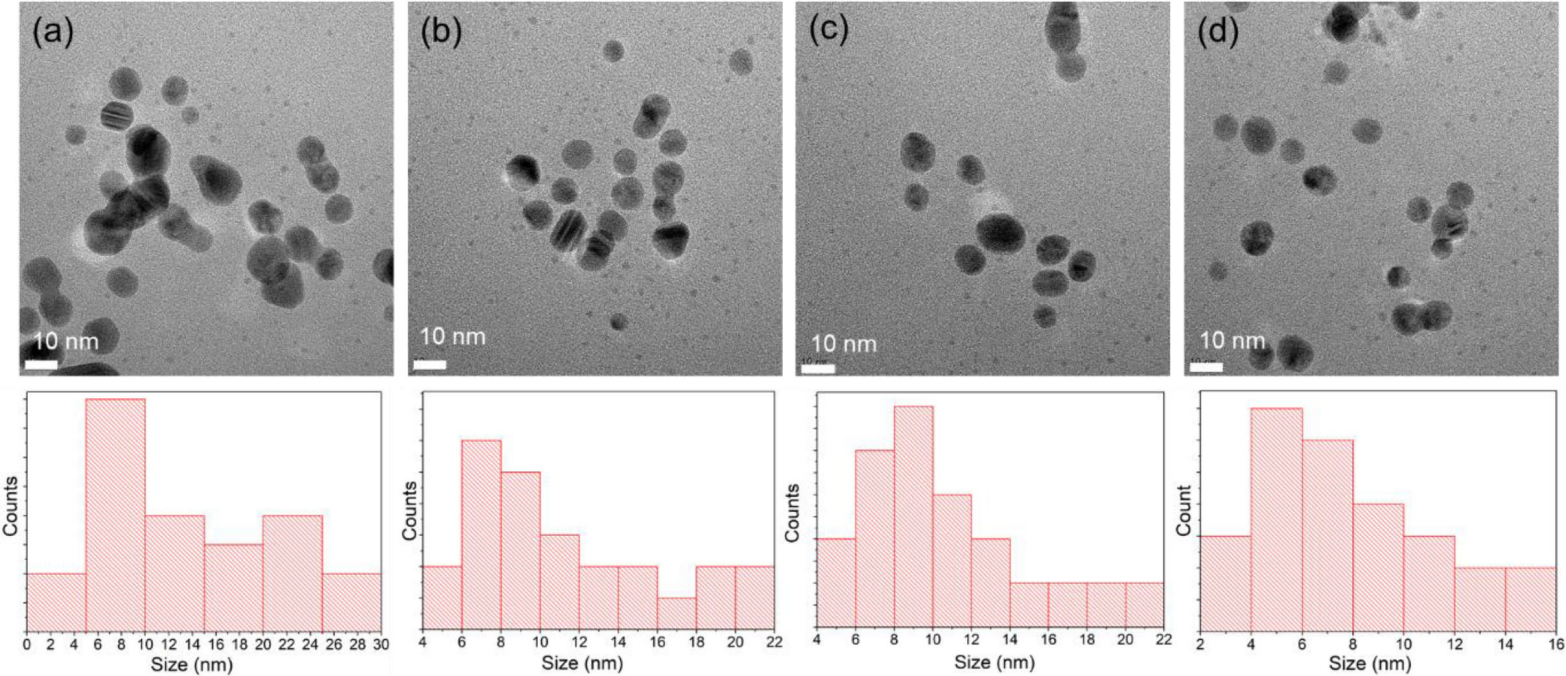
High-resolution transmission electron microscopy (HR-TEM) images and corresponding particle size distribution histograms illustrating the effect of NaOH concentration on the morphology, size distribution, and dispersion of AgNPs. AgNPs were synthesized using *Z. officinale* rhizome extract under alkaline conditions with NaOH concentrations of **(A)** 2 mM, **(B)** 3 mM, **(C)** 4 mM, and **(D)** 5 mM.

The effect of pH on AgNP size was further investigated in this green synthesis. The pH of the reaction mixture was controlled by varying the NaOH concentration (2–5 mM; **Figure 5**), and corresponding HRTEM images were obtained. Lower alkaline pH conditions (2 mM NaOH) resulted in a wide range of AgNP sizes, with evident signs of aggregation (**Figure 5A**), owing to slow nucleation and growth rates, thereby allowing AgNP formation with a broad size distribution. However, concentrations of NaOH (3–5 mM, high pH) resulted in the synthesis of small-sized AgNPs with good dispersion (**Figures 5B–D**) due to controlled nucleation followed by rapid growth, resulting in small-sized particles. The optimal NaOH concentrations are necessary to initiate the reduction of AgNO_3_, sufficiently stabilize the AgNPs, and prevent aggregation due to the high degree of deprotonation of biomolecules (Nishimura et al., 2011). These observations are also consistent with previous reports stating that alkaline reaction mixtures readily facilitate the synthesis of metal NPs and significantly influence the initial nucleation and growth stages of AgNP formation (Habibullah et al., 2021).

**Figure 6:**
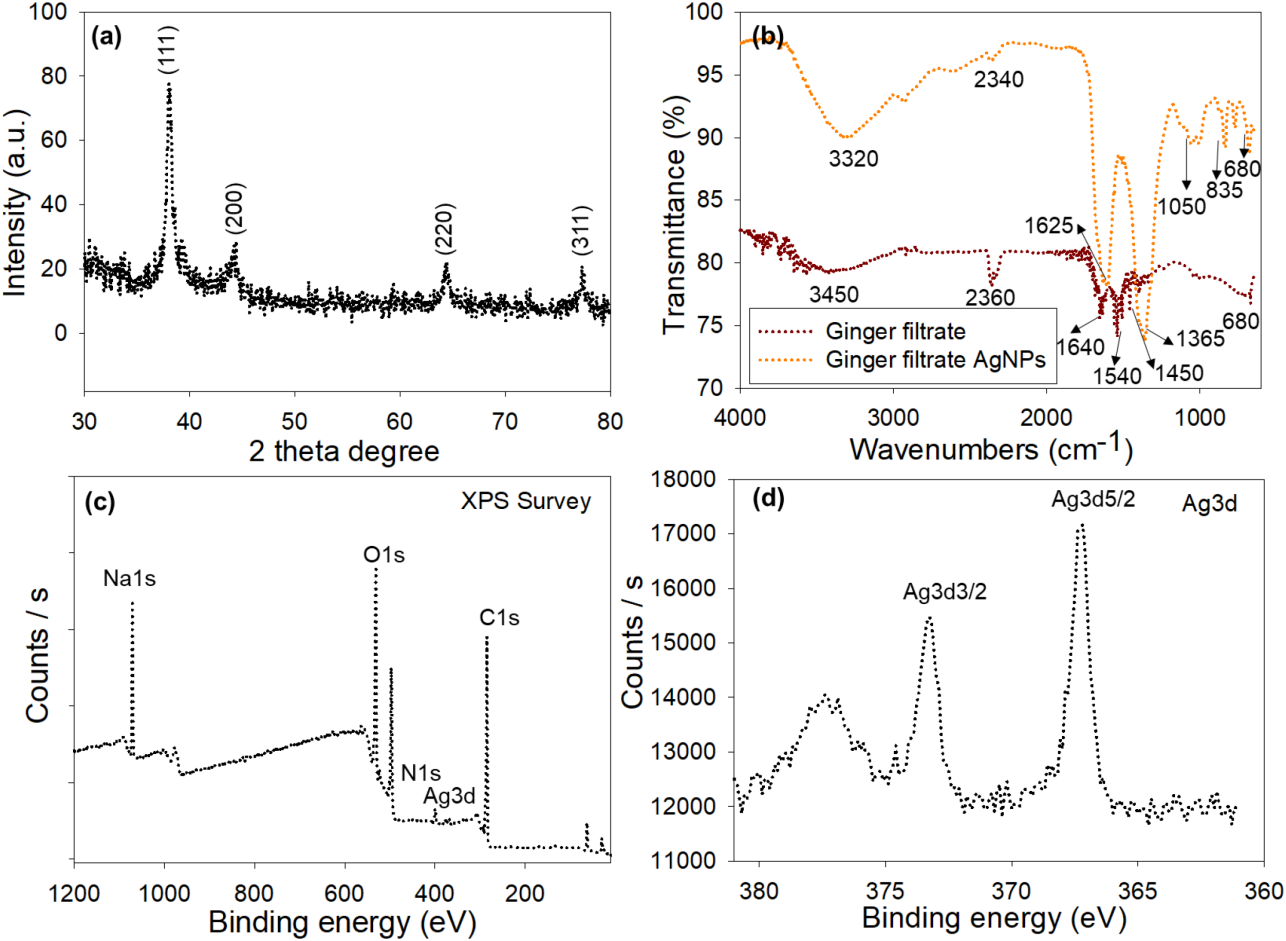
Characterization of biosynthesized AgNPs. **(A)** X-ray diffraction (XRD) patterns confirming the crystalline face-centered cubic (fcc) structure of metallic silver. **(B)** Fourier-transform infrared (FTIR) spectra indicating phytochemical functional groups from *Z. officinale* extract involved in the reduction of Ag^+^ ions and stabilization of AgNPs. **(C)** X-ray photoelectron spectroscopy (XPS) survey spectrum revealing the elemental composition. **(D)** High-resolution Ag 3d XPS spectrum confirming the metallic state of Ag^0^.

Selected-area electron diffraction (SAED) results confirm the crystalline nature of the AgNPs obtained by the aqueous extract method. In the SAED pattern, bright rings with distinct spots are clearly visible (**Supplementary Figure 2**), indicating that the obtained AgNPs are crystalline and have numerous orientations. Each ring consists of spots that demonstrate the crystalline character of the AgNPs and also the fcc structure of the AgNPs (Venugopal et al., 2017). The crystalline nature of the AgNPs synthesized was also examined by X-ray diffraction (XRD). AgNPs obtained by this method were characterized by XRD at a scanning speed of about 2°/min over the 2θ range of 30° to 80°. From the XRD pattern, clear peaks were observed for the 2θ values of 38.24°, 44.17°, 64.25°, and 77.35°, attributed to the (111), (200), (220), and (311) planes of the crystal structure of the zero-valent Ag, respectively (**Figure 6A**). These values are in agreement with the standard JCPDS values for the crystal structure of the Ag metal (Elumalai et al., 2014; Osonga et al., 2019). The broad peak at 2θ = 38.24° was used to determine the crystallite size with the Scherrer formula, yielding a range of 14 ± 6 nm, consistent with the desired size range for any green method. In addition, the high intensity of the (111) plane peak indicates that the crystal structure of the AgNPs is dominated by this plane, a feature of their nanoscale size, high crystallinity, and purity (Badán et al., 2022).

FTIR spectroscopy results showed that the functional groups present in the extract play a key role in the green synthesis and stabilization of AgNPs. The bands in the FTIR spectrum in the 4000–600 cm^–1^ region are: 3320–3340 cm^–1^ corresponds to –OH stretching in alcohols/phenolics that could have been responsible for reducing AgNO_3_ to AgNPs. Whereas the peak at 1625 cm^–1^ corresponds to amide I bands or C=O stretching in peptides, the peak at 1365 cm^–1^ corresponds to C–N stretching in amines/amides, and the band at 1050 cm^–1^ corresponds to C–O stretching in alcoholic compounds. However, additional bands at 850 cm^–1^ and 650 cm^–1^ correspond to C–H out-of-plane bending or in-plane bending in aromatic compounds. The presence of phenolic, aromatic, and alcoholic functional groups, among others, in the plant extract phytochemicals are confirmed by the FTIR spectrum (**Figure 6B**). Typically, 3450 cm^–1^ corresponds to O–H or N–H stretching in alcohols/phenolics or amines/amides in proteins; 1640 cm^–1^ corresponds to C=O stretching in peptides (Sanchez Ramirez et al., 2021), whereas the peaks n the range 1500–1600 cm^–1^ corresponds to Amide II bands in proteins/peptides ( (Chatterley et al., 2022). The absence of bands in the 850–650 cm^–1^ region corresponding to out-of-plane bending in aromatics or metal-oxygen stretching could be due to the interaction between the oxygen-containing compounds and the AgNP surface (Wang et al., 2023b).

The FTIR results were further validated by XPS to elucidate the functional groups interacting with the AgNP surfaces. The XPS results were also used to determine the elemental composition of AgNPs prepared by the extract method. The presence of different high-intensity XPS peaks for O1s, C1s, N1s, and Na1s, along with Ag3d peaks (**Figure 6C**), suggests that a group of biomolecules facilitates the reduction of AgNO_3_ and their possible interaction with AgNPs. The XPS peaks for the Ag3d region indicate the presence of metallic Ag in the NPs, as observed for the Ag 3d_5/2_ peak at 367.3 eV and the Ag 3d_3/2_ peak at 373.3 eV (**Figure 6D**), with a spin-energy split of about 6 eV, which agrees with the earlier report (Talabani et al., 2021).

**Figure 7:**
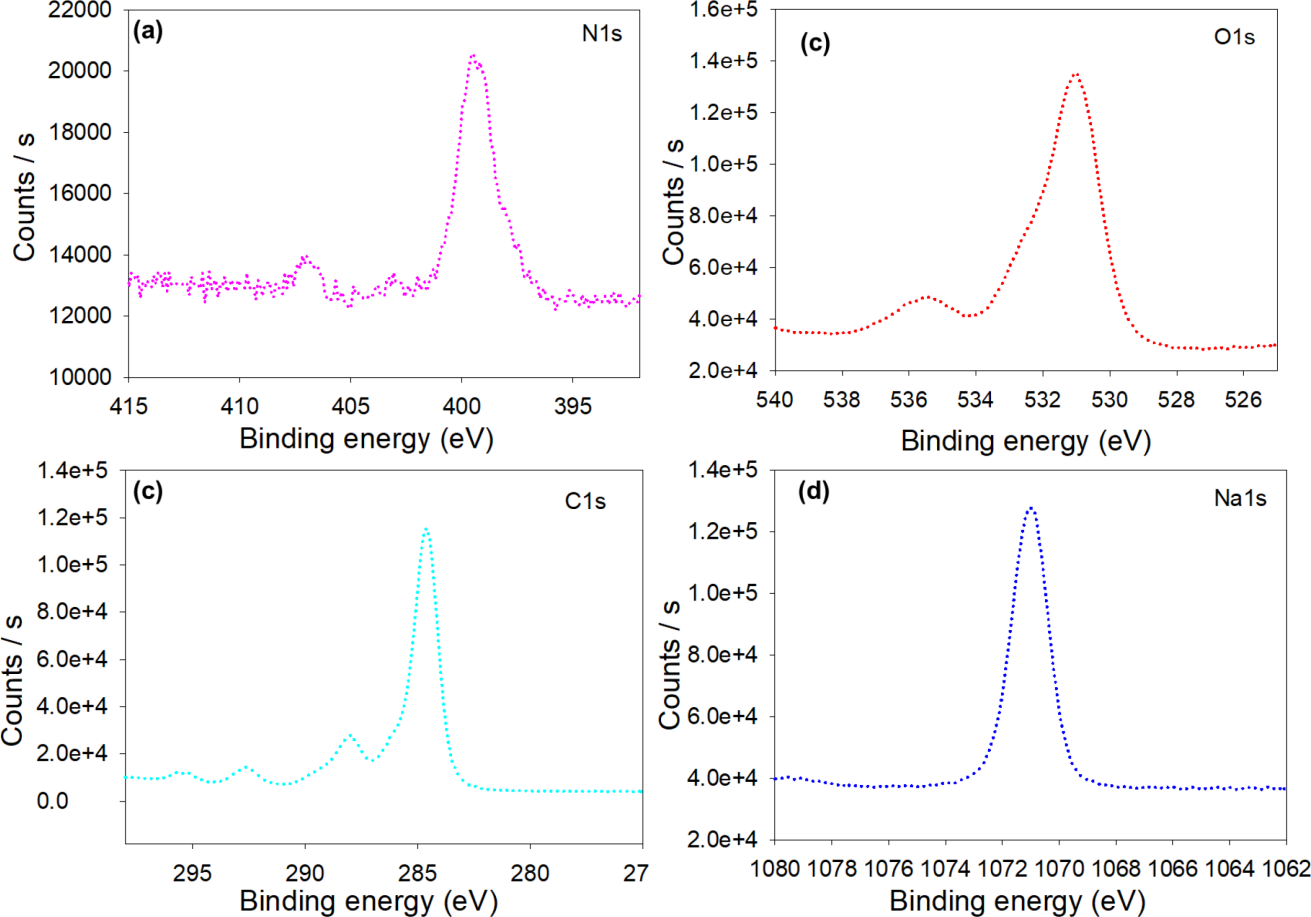
High-resolution XPS core-level spectra of surface-associated elements indicating phytochemical capping and stabilization of the biosynthesized AgNPs: **(A)** N 1s, **(B)** O 1s, **(C)** C 1s, and **(D)** Na 1s.

The N1s peak originates from nitrogen-containing compounds or proteins on the surface of AgNPs. The binding energies for these compounds were observed around 399.4 eV for amines and 402.9 eV for amides (**Figure 7A**). The O1s peak shows the presence of oxygen-containing groups with binding energies at around 530.9 and 535.4 eV, owing to hydroxyl and carbonyl groups, respectively (**Figure 7B**). The C1s peak was found at binding energy about 284.8 eV, attributes to C–C or C–H bonds, showing the involvement of sp2 or sp3 hybridized carbon (**Figure 7C**). The presence of a small peak at 287.9 eV is associated with C–O or C–N bonds, indicating phenol or amine groups in the biomolecules. Whereas a small peak at 292.6 eV suggests an oxidized carbon state, which could originate from C=O or O–C=O groups. However, the Na 1s peak at approximately 1071 eV is most likely due to residual Na ions (**Figure 7D**). These results confirm that surface chemistry and interactions between the biomolecules and the AgNPs control the properties of the metallic NPs (Ruíz-Baltazar et al., 2018). The present study further explores the multifunctional evaluation of biosynthesized AgNPs, including their antimicrobial and agricultural applications, as well as their ecotoxicological effects.

### 3.3 Antibacterial results

**Figure 8:**
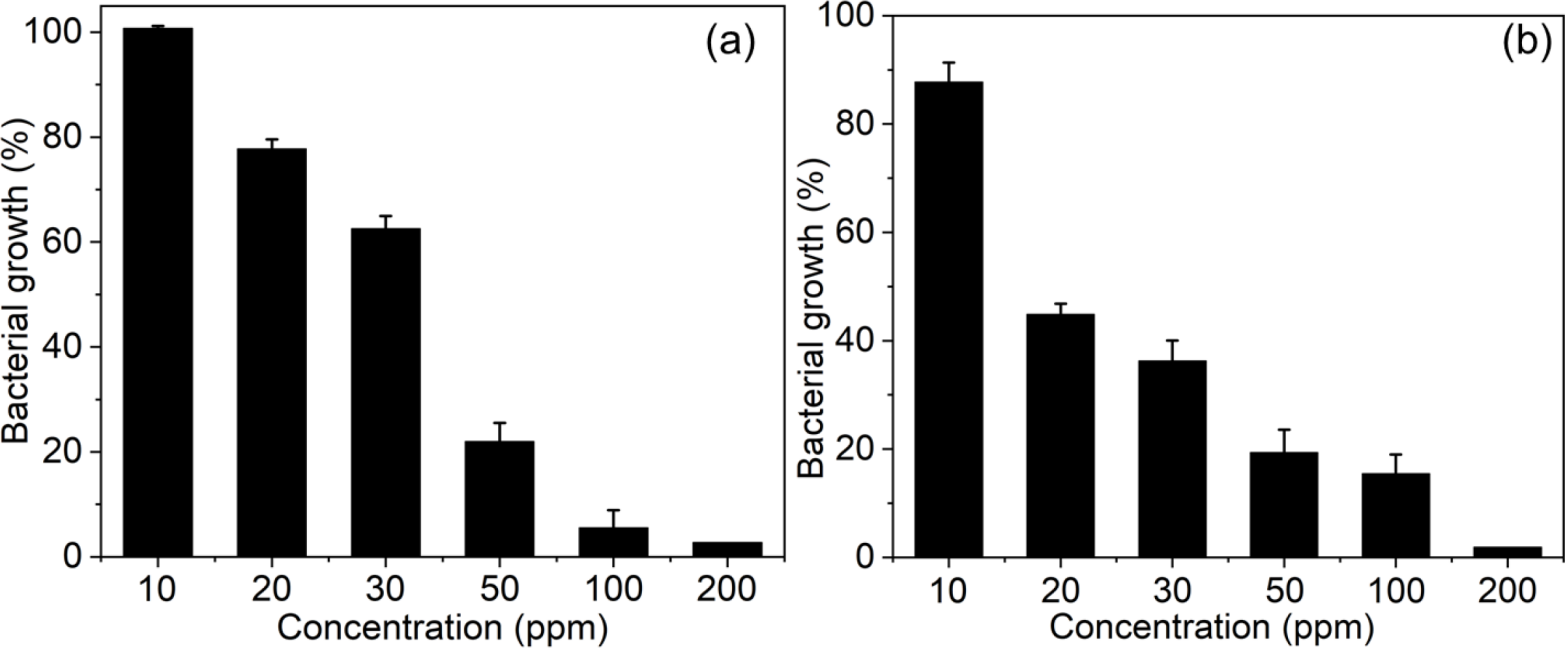
Concentration-dependent antibacterial activity of biosynthesized AgNPs (20–200 ppm) against **(A)** *S. aureus* and **(B)** *E. coli*, demonstrating progressive inhibition of bacterial growth with increasing nanoparticle concentration.

The antibacterial potential of AgNPs was tested against MDR strains of *S. aureus* and *E. coli* at concentrations ranging from 20 to 200 ppm. The result suggests that the antibacterial potential of AgNPs against MDR bacterial pathogens increased with increasing AgNP concentration (**Figure 8**). Furthermore, it underscores the importance of dosage in achieving the desired antibacterial properties. As shown in **Figures 8A**, the lethal concentration 80 (LC_80_) for *S. aureus* is about 50 ppm, which validates that 50 ppm or higher of AgNPs is essential to inhibit the growth of S. aureus effectively. Similarly, the LD_80_ for *E. coli* was also approximately 50 ppm, indicating that both Gram-positive and Gram-negative bacterial pathogens are susceptible to AgNPs. However, a significant decrease in bacterial viability, as indicated by an LD_50_, was observed for *E. coli* at a lower AgNP concentration of about 20 ppm (**Figure 8B**), indicating that Gram-negative bacteria are more susceptible to AgNPs, consistent with previous reports (Khalifa et al., 2025). The concentration-dependent reduction in bacterial viability indicates the potential of AgNPs as an alternative antibacterial agent against MDR bacterial strains (Loo et al., 2018).

To further investigate the bactericidal potential of AgNPs, colony-count assays were performed at varying concentrations (**Supplementary Figure 3**). This method allowed the evaluation of viable bacterial cells present in samples treated with different concentrations of AgNPs. The bactericidal activity of AgNPs is used to assess their potential to disinfect MDR bacterial strains. Furthermore, the results were used to confirm the achievement of total bactericidal activity at the effective concentration of the AgNPs. From the results, a minimum bactericidal concentration is about 100 ppm, sufficient to achieve complete bactericidal effects, eradicating both Gram-positive and Gram-negative bacterial cells (**Supplementary Figures 3A, B**). Thus, AgNPs have potential applications in eliminating bacterial biofilms and providing insights into their complete bactericidal efficacy; such information could be used to formulate the dosage for potential therapeutic applications (Dakal et al., 2016).

To investigate morphological changes in AgNP-treated bacteria, *S. aureus* and *E. coli* were exposed to 50 ppm AgNPs and then subjected to Gram staining and FE-SEM analysis. At this concentration, cell wall structural irregularities were observed, including large aggregates of cells and debris, indicating the disruptive effect of AgNPs. Compared with the non-treated cells (**Supplementary Figures 4A,B**), the reduction in stain intensity may be due to compromised cell wall and membrane integrity resulting from interaction with AgNPs. The pale blue and yellow colors may be due to reduced staining by residual cell material from *S. aureus* and *E. coli*, respectively (**Supplementary Figures 4C,D**). Such color changes observed in both *S. aureus* and *E. coli* were attributed to impaired cellular components that vary in their Gram-staining efficacy, as suggested in a previous report (Kim et al., 2024). It also provides indications of the physical damage-related mechanism of action of AgNPs. The formation of aggregates and debris also suggests cell death, possibly via cell-pore formation and loss of membrane permeability, eventually leading to cell lysis. Surface-level optical microscopy is limited in its ability to validate such claims; bacterial cell wall interactions with AgNPs were further examined using FE-SEM.

The FE-SEM imaging technique was further applied to elucidate the antimicrobial mechanism of action of the synthesized AgNPs. The morphological changes and cell debris were observed, demonstrating the critical role of AgNPs against bacterial pathogens and their potential to mitigate biofilm development in MDR bacterial pathogens. The control samples were used to show the original morphology of bacterial cells for both *S. aureus* and *E. coli* (**Supplementary Figures 5A, B**). The morphology of *S. aureus* and *E. coli* remained intact, with undistorted cell walls and smooth cell walls and membranes, confirming the healthy cell morphology shown in **Supplementary Figures 5A, B**.

**Figure 9:**
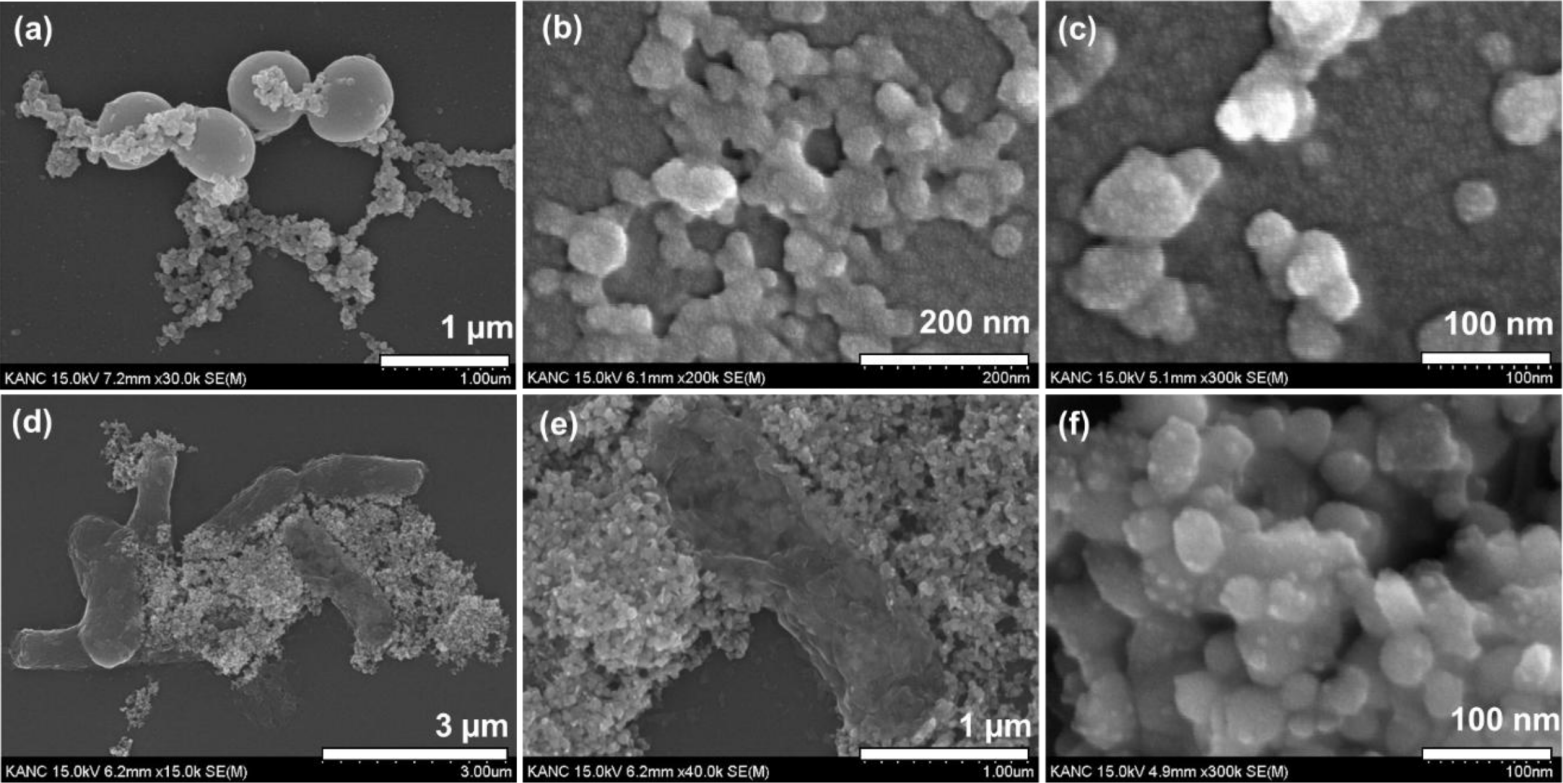
Field-emission scanning electron microscopy (FE-SEM) micrographs at multiple magnifications revealing severe membrane damage, surface collapse, and cellular lysis in bacteria following treatment with 50 ppm AgNPs: **(a–c)** *S. aureus* and **(d–f)** *E. coli*.

Based on the comparison with the reference images, the figures show notable changes observed after treatment with AgNPs at 50 ppm. After exposing *S. aureus* and *E. coli* cells to AgNPs, significant morphological changes were observed (**Figure 9**). These images highlight the potent bactericidal activity of AgNPs and cause severe morphological damage in *S. aureus* cells, as shown in **Figures 9A–C**. Similar to changes in cell size and morphology resulting from leakage of cell contents, as shown in the images, and attributed to changes in osmotic balance in *S. aureus* cells (Liao et al., 2019). Likewise, untreated *E. coli* cells display a characteristic rod-shaped morphology with a smooth outer membrane (**Supplementary Figure 5B**), whereas AgNP-treated *E. coli* cells exhibit pronounced structural alterations, including deformation, membrane fractures, and surface roughening (**Figures 9D–F**). The membranes of both *S. aureus* and *E. coli* cells were clearly evident, with compromised cell wall structures, exhibiting irregular boundaries and cracks, among other forms of damage. The damage also implies disruption or interaction of AgNPs with the cell envelope, leading to irregular, rough structural deformities in cell morphology (Dakal et al., 2016). These observations are consistent with the proposed mechanism of action of AgNPs, which involves disruption of the bacterial cell membrane, ultimately leading to bacterial cell death (Mikhailova, 2020; Yan et al., 2018).

### 3.4 Seed priming results

In the present study, we assessed the effects of AgNPs on seed germination, growth, and biomass yield of *Trigonella foenum-graecum* (fenugreek) and *Vigna radiata* (mung bean) under salt stress conditions. The present study was designed to investigate the role of AgNPs in improving seed germination under salt stress. For this purpose, AgNPs at 20, 30, 40, 50, and 60 ppm were applied as treatments, and pristine seeds and hydropriming with DI water were used as control treatments. Seeds were germinated for 24 to 48 hours to assess differences in germination rates. The percentage of seed germination ranged from 98% ± 3 for both seed types across all control and experimental samples (**Supplementary Figure 6**, **S7**). Radicle formation was also observed for 36 hours, indicating embryonic root development. This is shown in the **Supplementary Figure 6**, **S7**.

However, there were no significant differences in seed germination between AgNP-treated seeds and pristine seeds. AgNP application successfully decreased the mean germination time and increased the germination rate, indicating that seed priming could play an important role in improving seed germination and seedling growth (Chandel et al., 2024; Khan et al., 2023).

The results showed a considerable improvement in the development of the embryonic roots of mung bean seedlings, especially at AgNP concentrations of 30 and 40 ppm (**Supplementary Figures 6A–G**). Whereas increased AgNPs concentration of about 50 to 60 ppm showed the no negative effect in embryonic root development for mung bean seedlings. However, the results shown in the supplementary (**Supplementary Figures 7A–G**) showed a substantial increase in the development of embryonic roots for fenugreek seedlings treated with all concentrations of AgNPs. The marked improvement in the embryonic root development of fenugreek seedlings indicates that AgNP priming could be a useful tool for enhancing crop growth, especially under stress (Lin and Xing, 2007). In another research, ZnO NMs were tested to improve tomato seedling growth and mitigate the negative effects of salt stress (Hanif et al., 2023). This observation is consistent with results from treating seeds with AgNPs, which improved radicle and plumule emergence (Acharya et al., 2020). Thus, AgNPs-based seed priming is beneficial to agricultural applications, as it could lead to the elimination of conventional agrochemicals and promote alternative seed treatment approaches (Ansari et al., 2023; Kim et al., 2024; Parveen and Rao, 2015).

**Figure 10:**
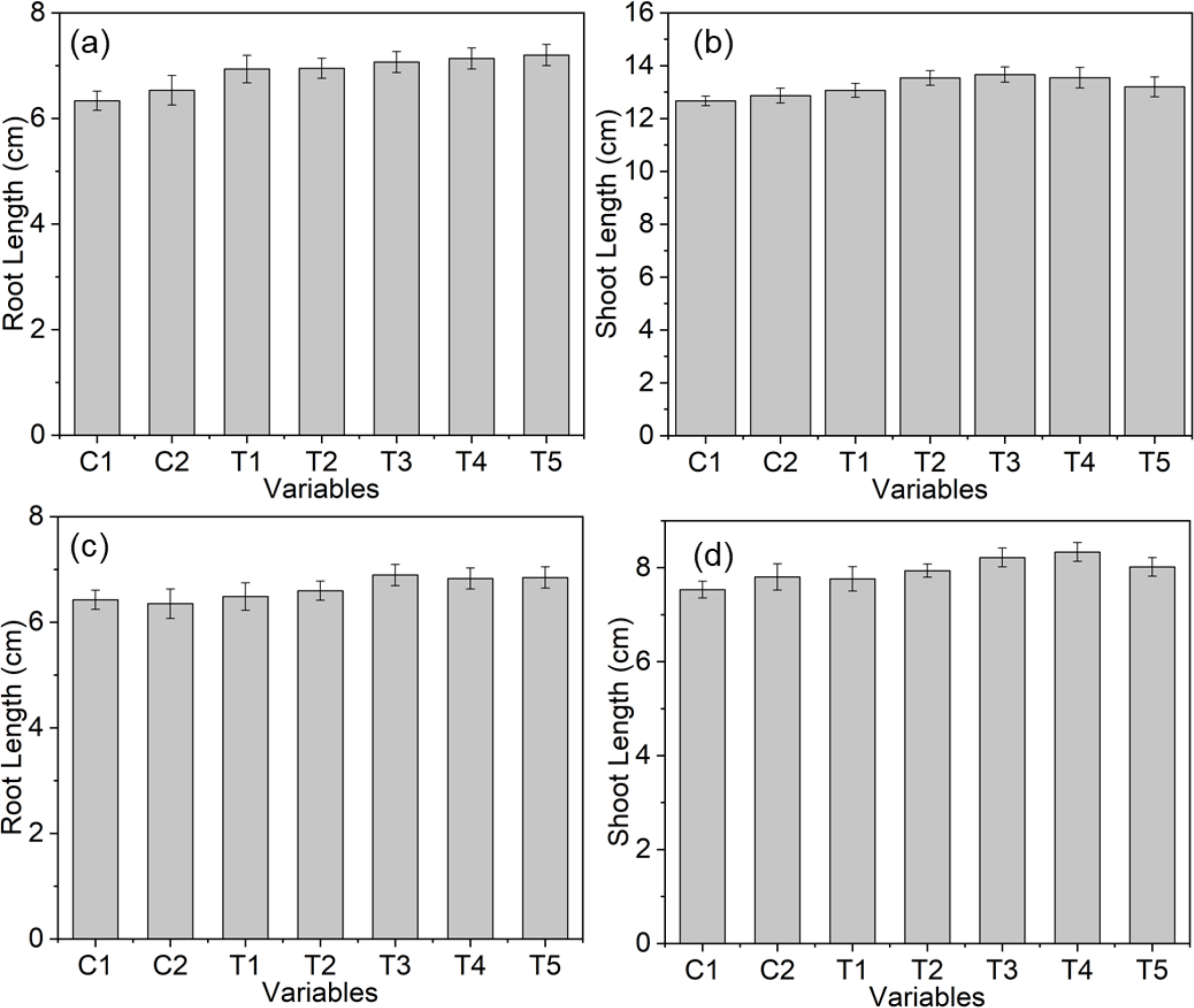
Effect of AgNP-mediated seed priming on early seedling development. Quantitative comparison of root and shoot lengths of **(A, B)** *Vigna radiata* and **(C, D)** *Trigonella foenum-graecum* under different AgNPs concentrations (T1–T5: 20–60 ppm) relative to non-primed and hydro-primed controls, demonstrating enhanced germination and growth performance.

To evaluate the seed-priming applications of AgNPs at 20 to 60 ppm, the growth parameters of the seedlings, such as shoot and root lengths, were measured in centimeters on the 5^th^ days of germination (**Figure 10**). The AgNP-primed seedlings showed higher shoot and root lengths than the non-primed control seedlings, confirming the positive impact on the growth of both the tested crops. However, there was a clear trend of improved growth with increasing AgNPs concentration. It is important to note that no stunted growth was observed in the lengths of roots and shoots treated with the maximum concentration (AgNPs, 60 ppm), indicating healthy growth throughout the experiment (**Figure 10**). An elongation-promoting effect was not statistically significant, however, increase in the lengths of roots and shoots in mung bean been observed (**Figures 10A,B**). Similar findings were observed in fenugreek seedlings, in which AgNP priming enhanced shoot and root development compared with the non-primed and hydro-primed controls. A visible increasing trend in the lengths of both roots and shoots of fenugreek seedlings was observed across all tested AgNPs concentrations, suggesting improved growth compared to control seedlings (**Figures 10C,D**). More importantly, there was no observable phytotoxicity in morphology, growth, root length, or leaf discoloration across all tested AgNPs concentration ranges (**Supplementary Figures 8A,B**), suggesting safe application, especially for tested concentrations (Aslani et al., 2014; Gupta et al., 2018). This kind of positive result could be attributed to improved water uptake, enzyme activity, reactive oxygen species, and increased metabolic rates during germination (Li et al., 2024; Mahakham et al., 2017; Mehmood, 2018; Nayeri et al., 2023; Nile et al., 2022; Wang et al., 2023a).

Thus, seed priming with AgNPs at appropriate concentrations can be an effective approach to enhance seedling performance, offering nanotechnology-assisted option in agriculture. This can serve as an alternative method for improving seed and plant growth and disease management, ultimately enhancing crop production (Lv et al., 2023; Soni et al., 2024; Trzcińska-Wencel et al., 2023). Literature on improved growth of seedlings after treatment with NMs attributed to the rapid division and extension of plant cells and promoting the development of shoots and roots (Guzmán-Báez et al., 2021; Syu et al., 2014; Wang et al., 2020). Future studies should focus on conducting field-level research on the applications of AgNPs and other NMs in seed priming and on understanding the molecular and physiological basis of these benefits. Such studies needed to validate the safety, effectiveness, and feasibility of NMs in seed priming, paving the way for the safe use of nanotechnology-assisted agricultural products (Gade et al., 2023; Ijaz et al., 2023; Tomaszewska-Sowa et al., 2023; Zhang et al., 2022).

### 3.5 Zebrafish embryo toxicity studies

The toxicity of AgNPs was evaluated using zebrafish (*Danio rerio*) embryos as a model organism. Zebrafish embryos have emerged as an alternative for ecotoxicological studies because of their high sensitivity to toxicants, rapid development, transparent embryos, and close genetic and physiological similarities to higher vertebrates (Michaelis et al., 2025). In this study, the toxicity of biosynthesized AgNPs was evaluated by exposing zebrafish embryos to two concentrations (10 ppm and 20 ppm) and comparing them with untreated embryos. The observations were made at specific intervals (24, 48, and 72 hours post-fertilization [hpf]) and at specific endpoints. Parameters included survival rate, hatching rate, and morphological and physiological changes, which were recorded as shown in **Table 1**.

**Table 1:** Hatching and survival rates of zebrafish embryo and larvae (24–72 hpf) exposed to different concentrations of AgNPs.

Sr. no	Group	Hours (hpf)	Hatching rate	Hatching rate (%)	Survival rate (no.)	Survival rate %
1	Control	24	0 ± 0.0	0 ± 0.0	0 ± 0.0	0 ± 0.0
		48	30 ± 0.0	83.33 ± 6.89	30 ± 0.0	100 ± 0.0
		72	6 ± 0.0	100 ± 0.0	30 ± 0.0	100 ± 0.0
2	Ag NPs (10 ppm)	24	0 ± 0.0	0 ± 0.0	15 ± 2.74	50 ± 9.13
		48	6 ± 2.19	20 ± 7.30	10 ± 2.58	33.33 ± 8.60
		72	0 ± 0.0	0 ± 0.0	0 ± 0.0	0 ± 0.0
3	Ag NPs (20 ppm)	24	0 ± 0.0	0 ± 0.0	8 ± 2.42	26.66 ± 8.08
		48	2 ± 1.41	6.66 ± 4.55	2 ± 1.41	6.66 ± 4.55
		72	0 ± 0.0	0 ± 0.0	0 ± 0.0	0 ± 0.0

In the control group, embryonic development remained normal throughout the experimental period. At 48 hpf, hatching occurred in 30 embryos, whereas the remaining embryos successfully hatched at 72 hpf, yielding a hatching percentage of about 100%. In contrast, the embryos treated with AgNPs showed significant toxicity. The survival rate at 10 ppm decreased to 50% by 24 hpf. There were only 6 embryos hatched at 48 hpf, with the hatching rate of about 20% and survival rate of about 33%, whereas at 72 hpf, there was complete mortality with no surviving embryos. The toxic effects were more pronounced at a higher concentration of 20 ppm, with a survival rate of 26% at 24 hpf. Later, at 48 hpf, only 2 embryos hatched, indicating significant toxicity, with hatching and survival rates of about 6% each. Similarly, a complete mortality was observed, with no surviving embryos at 72 hpf. The data indicate that AgNPs exhibit time- and dose-dependent toxicity and interfere with embryonic development in zebrafish. This study has shown that even relatively low concentrations of AgNPs can pose significant developmental toxicity in zebrafish embryos (Cheng et al., 2025), thereby highlighting the potential ecological risks posed in aquatic environments (Wāng et al., 2024). A recent report examined various types of NMs and their effects on zebrafish across multiple biological levels, highlighting developmental abnormalities, altered gene expression, and serious toxicological concerns (Mutalik et al., 2024).

The toxicity of AgNPs was evaluated by measuring two important physiological parameters in zebrafish embryos after 48 hpf, and the results are presented in **Table 2**. The embryos in the control group showed two flicks per minute and an average heart rate of 116 beats per minute. However, in embryos treated with 10 ppm and 20 ppm Ag NPs, flicking ceased, and heart rates decreased to 78 and 48 beats per minute, respectively. This shows that AgNPs induce physiological toxicity in zebrafish embryos, affecting their neuromuscular and cardiac functions at low AgNP doses. The effects observed in this study could include oxidative stress, cellular damage, and ion channel imbalance, arising from interference by AgNPs and the release of Ag^+^ ions (Xu et al., 2018).

**Table 2:** Tail flick frequency and heart rates of zebrafish embryo and larvae 48 hpf after exposure to different concentrations of Ag NPs.

Sr. no	Group	Number of tail flicks/min	Heart beats/min
1	Control	2 ± 0.5	116 ± 9.5
2	Ag NPs 10 ppm	0 ± 0.0	78 ± 7.3
3	Ag NPs 20 ppm	0 ± 0.0	48 ± 4.6

**Figure 11:**
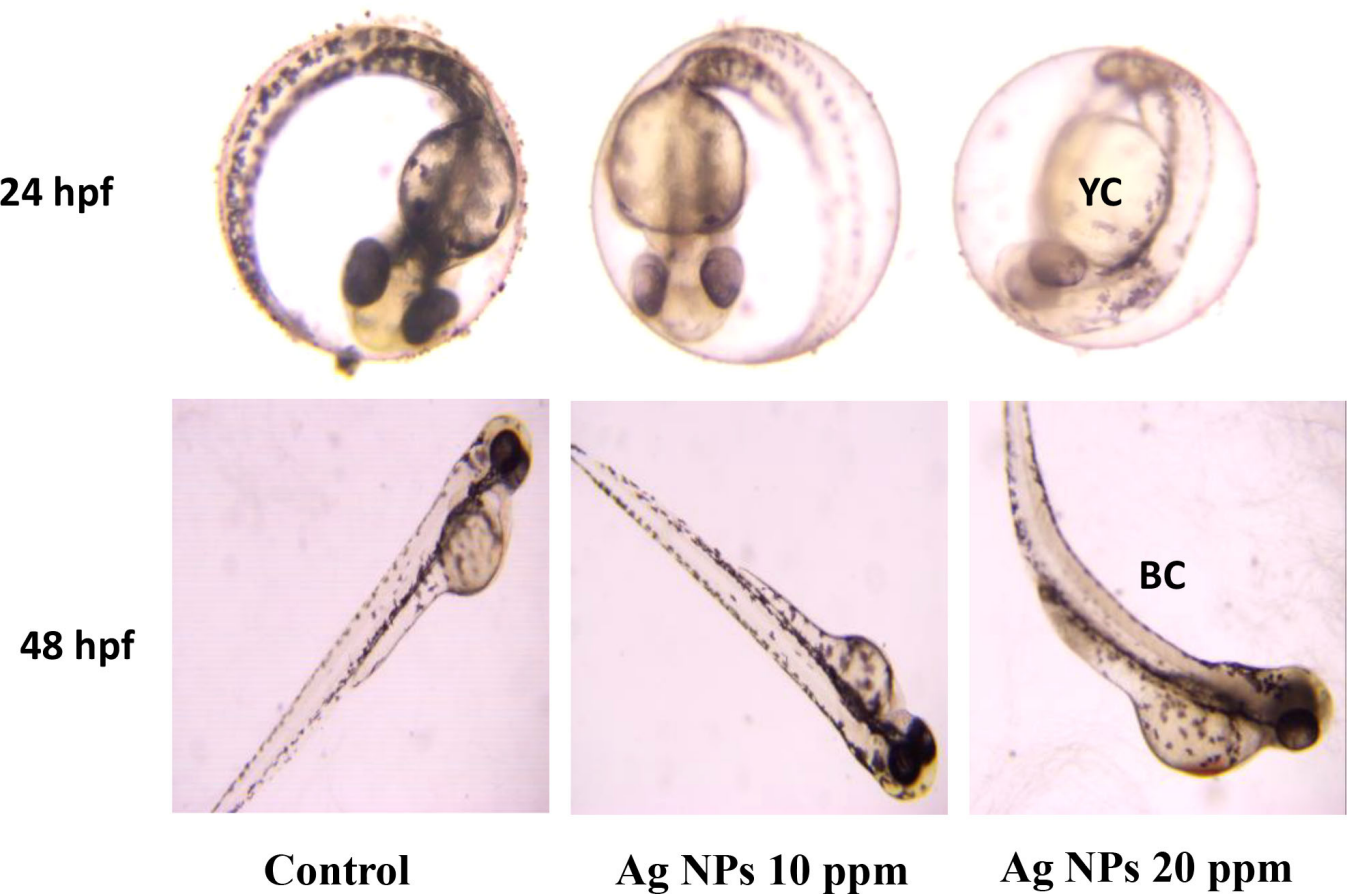
Representative images of zebrafish (*Danio rerio*) embryos and larvae (24–72 hpf) exposed to Ag NPs (10 and 20 ppm), showing dose-dependent developmental abnormalities, including yolk sac (YC) deformation and body curvature (BC), indicative of nanoparticle-induced embryo toxicity.

It was found that AgNP-treated embryos exhibited clear, pronounced morphological changes compared to the untreated control group. All these changes indicate that developmental toxicity was caused at a significant level due to the presence of AgNPs. The morphological changes in embryos exposed to AgNPs indicate that they disrupt normal embryonic development, affecting other important physiological activities during growth. The images obtained show morphological changes in embryos exposed to AgNPs. As shown in **Figure 11**, no hatching was observed in embryos exposed to AgNPs or in the control embryos at 24 hpf, which is consistent with the normal course of zebrafish development. However, further development in embryos exposed to AgNPs proceeded more slowly than in control embryos (**Figure 11**). Numerous morphological abnormalities observed in the embryos exposed to AgNPs included pericardial edema, yolk sac abnormalities, reduced body size, and noticeable developmental delay. Such toxicity may result from various factors, particularly oxidative stress, alterations in homeostasis, cardiac dysfunction, and interference with physiological processes during zebrafish embryonic development (Lu et al., 2024).

## 4 Risk-benefit considerations and prospects for AgNPs application

Potential applications of AgNPs in antimicrobial formulations and agriculture necessitate an in-depth analysis of the beneficial effects of AgNP exposure and the associated environmental risks (Ferdous and Nemmar, 2020). This study highlights the significance of concentration-dependent activities and functionalities of AgNPs, including antibacterial and seed-priming applications, and the use of toxicity thresholds in zebrafish embryos to ensure benefits and minimize ecological risks. Moreover, the antibacterial activity of AgNP was observed, indicating that exposure at an LD_80_ of 50 ppm effectively inhibits the growth of MDR bacterial strains. The results of the present investigation have also shown that AgNP exposure at 20–60 ppm enhances seed germination and seedling growth, which indicates the possible application of AgNP as a seed priming agent. Furthermore, optimizing the dose, monitoring environmental conditions, and understanding the fate and transport of NPs in natural environments will be vital to the effective and safe application of NPs. Zebrafish embryo toxicity assays have shown that exposure to 10–20 ppm AgNP is highly toxic to zebrafish at developmental stages, indicating that exposure at lower concentrations is harmful to aquatic organisms. Future studies need to emphasize on ecotoxicity, mechanistic studies of NP interactions with biological systems, and validation of safe and efficient applications. In addition, more advanced techniques, including interaction studies, proteomics, and environmental fate modeling, could provide greater insight into interactions with biological systems and their environmental fate. Such studies will be vital to the formulation of guidelines for regulating the development, commercialization, and use of NP-based products, especially in the agricultural and biomedical fields.

## 5 Conclusions

Green synthesis of AgNPs using *Z. officinale* rhizome extract is an eco-friendly, economically viable, and facile method that aligns with green chemistry. The optimized, safer synthesis method has shown promising results for producing AgNPs with precise particle size, shape, and surface properties. The antibacterial potential of the biosynthesized AgNPs was observed against MDR bacterial strains, including *S. aureus* and *E. coli*. Significant disruption of bacterial cell structures clearly indicates the potential of AgNPs as an alternative antibacterial agent. Moreover, AgNPs used for seed priming positively affected seedling growth, increasing seed germination, reducing mean germination time, and enhancing seedling vigor, indicating potential applications in medicine and agriculture. However, in the case where a zebrafish embryo toxicity test was conducted, it is clear that there is a dose and time-dependent developmental toxicity after exposure to AgNPs. Furthermore, significant negative effects, including lower survival rates, morphological abnormalities, and neuromuscular and cardiac dysfunction, were evident in embryos exposed to AgNPs. Therefore, it is important to fine-tune AgNP concentrations and assess potential ecotoxicological risks before large-scale applications. Nevertheless, there is a need to balance the benefits of AgNPs in medicine and agriculture against their safety concerns for humans and the environment. Future studies should focus on dose optimization, mechanistic studies, and field-level validation to ensure the sustainable and safe application of nanotechnology-based solutions.

## Data Availability

The original contributions presented in the study are included in the article/**Supplementary Material**. Further inquiries can be directed to the corresponding author.
