# Supplementary material for: Greener synthesis of silver nanoparticles from Zingiber officinale rhizome extract for multidrug-resistant pathogen control, seed vigor enhancement, and fish embryo toxicity assessment
Source: Front Cell Infect Microbiol. 2026 May 8;16:1798925. doi: 10.3389/fcimb.2026.1798925 (PMC13194146; doi:10.3389/fcimb.2026.1798925)
Supplement: Supplementary file 1 [file Supplementaryfile1.docx]

**Greener Synthesis of Silver Nanoparticles from *Zingiber officinale* Rhizome Extract for Multidrug-Resistant Pathogen Control, Seed Vigor Enhancement, and Fish Embryo Toxicity Assessment**

**Running Title: Green Silver Nanoparticles: Antimicrobial, Seed Priming, and Ecotoxicological Evaluation**

**Gajanan Sampatrao Ghodake^1^, Min Kim^2^, Jung-Suk Sung^2^, Karthikeyan Chandrasekaran^2^, Sandip Patil^3,4^, Vini Mehta^5^, Asad Syed^6^, Ali H. Bahkali^6^, and Dae-Young Kim^1^***

^1^Department of Biological and Convergent Environmental Science, Dongguk University-Seoul, 32 Dongguk-ro, Ilsanadong-gu, Goyang-si 10326, Gyeonggi-do, South Korea

^2^Department of Life Science, Dongguk University-Seoul, 32 Dongguk-ro, Ilsanadong-gu, Goyang-si 10326, Gyeonggi-do, South Korea

^3^Department of Hematology and Oncology, Shenzhen Children’s Hospital, Shenzhen, 518038, China.

^4^Paediatric Research Institute, Shenzhen Children’s Hospital, Shenzhen, 518038, China

^5^Global Research Cell, Dr. D. Y. Patil Dental College & Hospital, Dr. D. Y. Patil Vidyapeeth (Deemed to be University), Pimpri, Pune 411018, India

^6^Department of Botany and Microbiology, College of Science, King Saud University, P.O. Box 2455, Riyadh, 11451, Saudi Arabia

***Correspondence:** Dae-Young Kim; E-mail: sbpkim@dongguk.edu; Tel: +82-31-961-5122; Fax: +82-31-961-5122


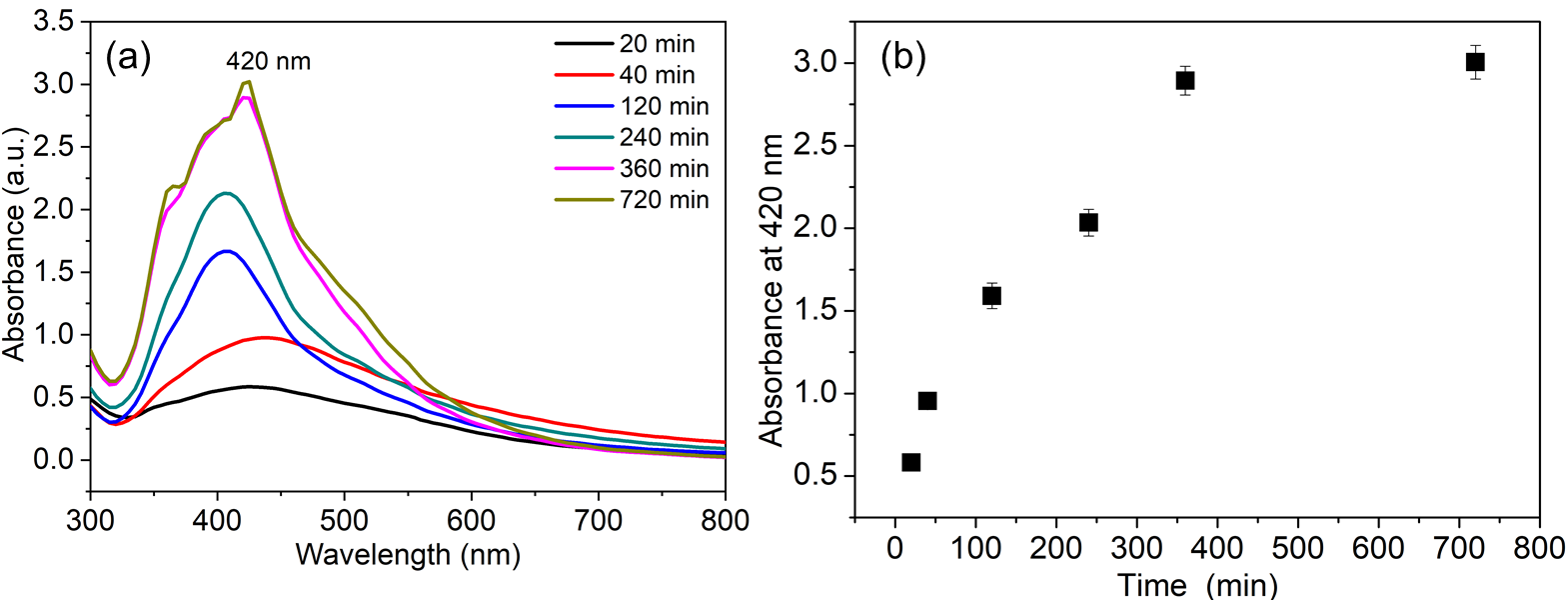


**Fig. S1.** The temporal evolution of surface plasmon resonance of biosynthesized AgNPs: (a) UV-Vis spectra illustrating the changes in spectral characteristics associated with AgNPs formation over increasing reaction time, (b) Absorbance intensity at 420 nm showcasing the yield of AgNPs.


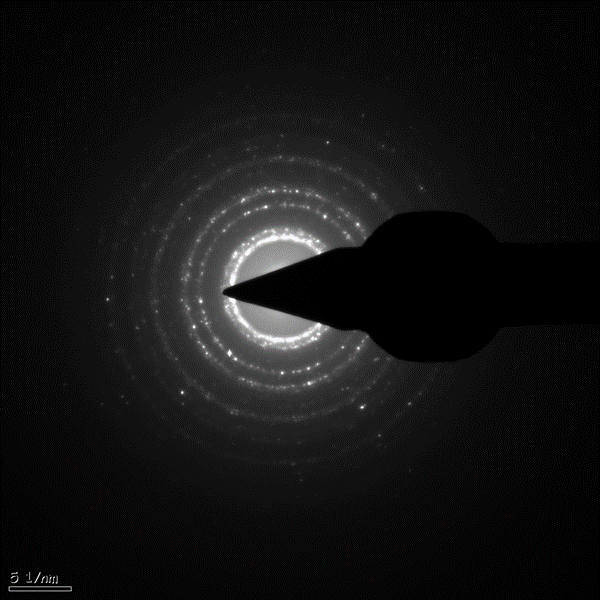


**Fig. S2.** Selected area electron diffraction pattern of AgNPs prepared using *Z. officinale* rhizome filtrate method reveals crystalline nature.


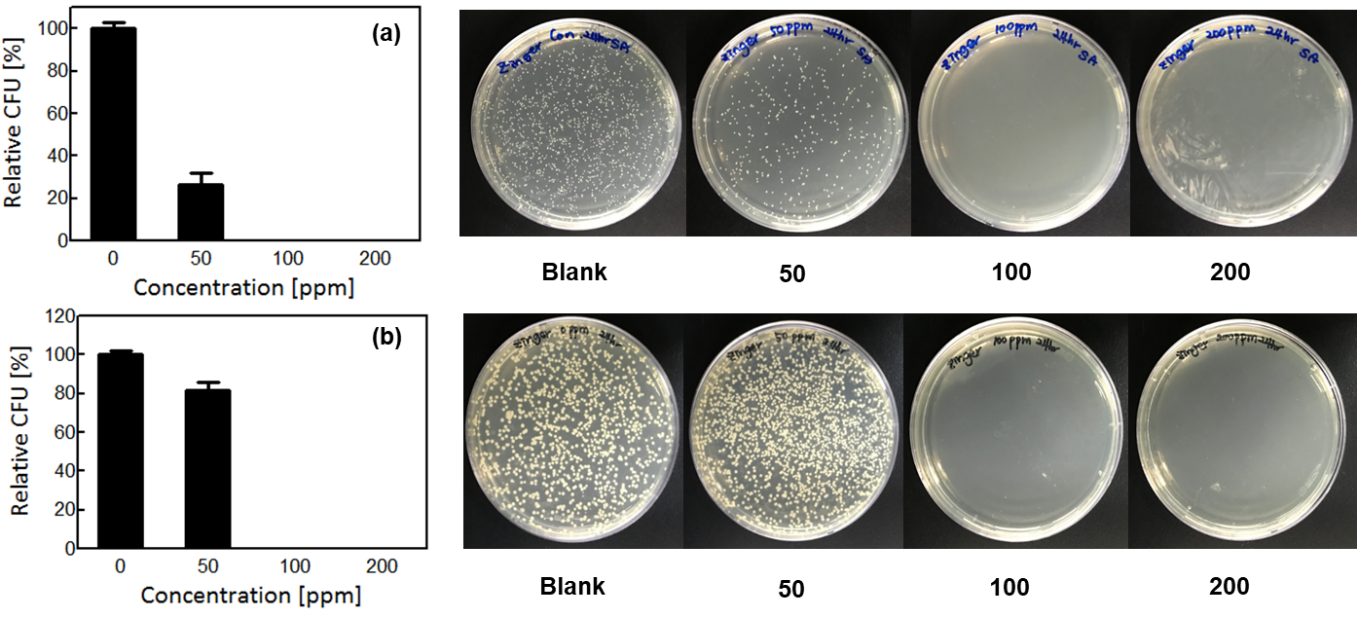


**Fig. S3.** Evaluation of the bactericidal activity of AgNPs using colony count method: (a) The colony forming units of *S. aureus* with varying concentrations of AgNPs, (b) The colony forming units of *E. coli* as a function of AgNPs concentration.


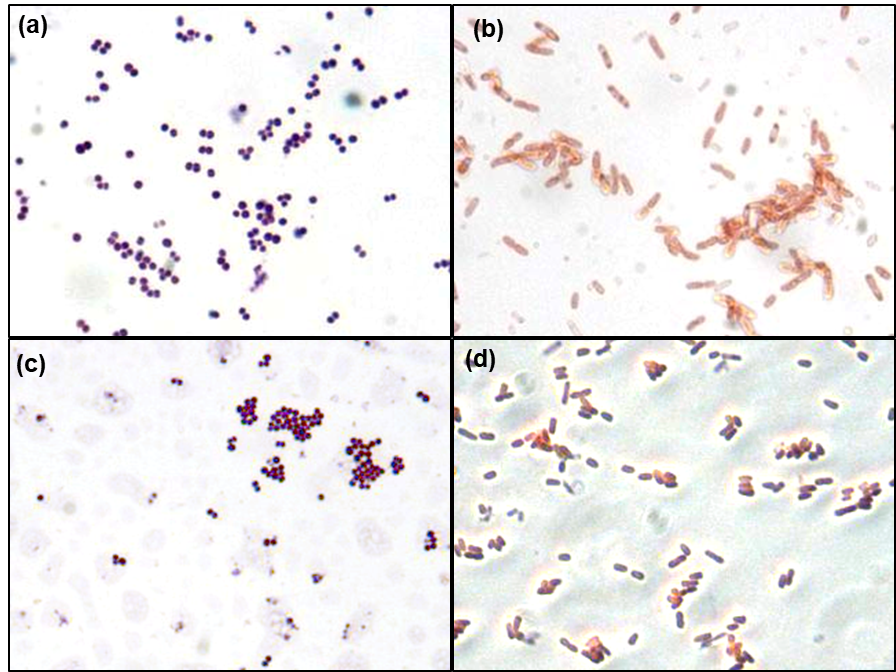


**Fig. S4.** Gram staining and light microscopy images illustrating AgNP-induced morphological damage in bacterial cells. Untreated controls of (a) *S. aureus* and (b) *E. coli* are compared with cells exposed to 50 ppm AgNPs showing structural disruption and cellular deformation in (c) *S. aureus* and (d) *E. coli*.


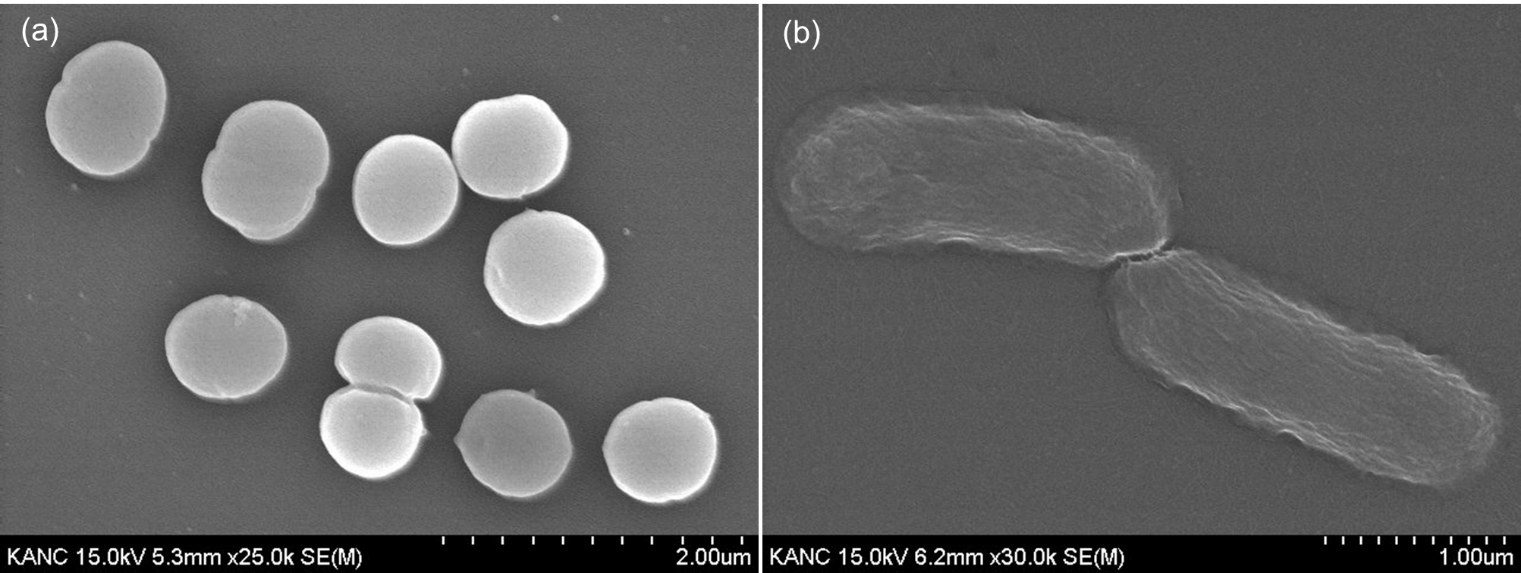


**Fig. S5.** FE-SEM imaging illustrating morphological integrity in bacterial cells: Control samples showcasing (a) the characteristic spherical morphology of *S. aureus* and (b) the typical rod-like structure of *E. coli*, evidencing the unaltered cellular morphology before AgNP exposure.


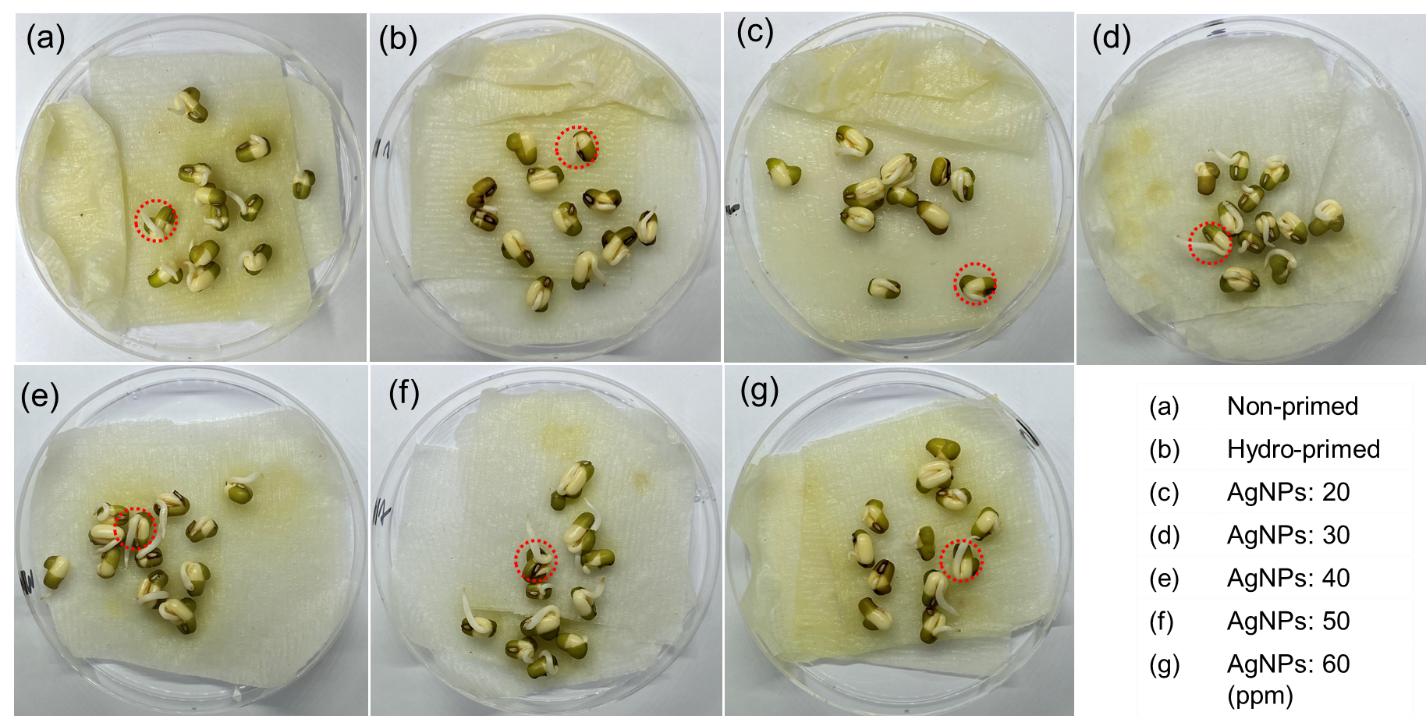


**Fig. S6.** Effect of seed priming with an aqueous dispersion of silver nanoparticles (AgNPs) on the germination of *Vigna radiata* seeds, observed at 36 hours post-treatment. The figure illustrates the comparative germination rates between seeds treated with AgNPs and control groups.


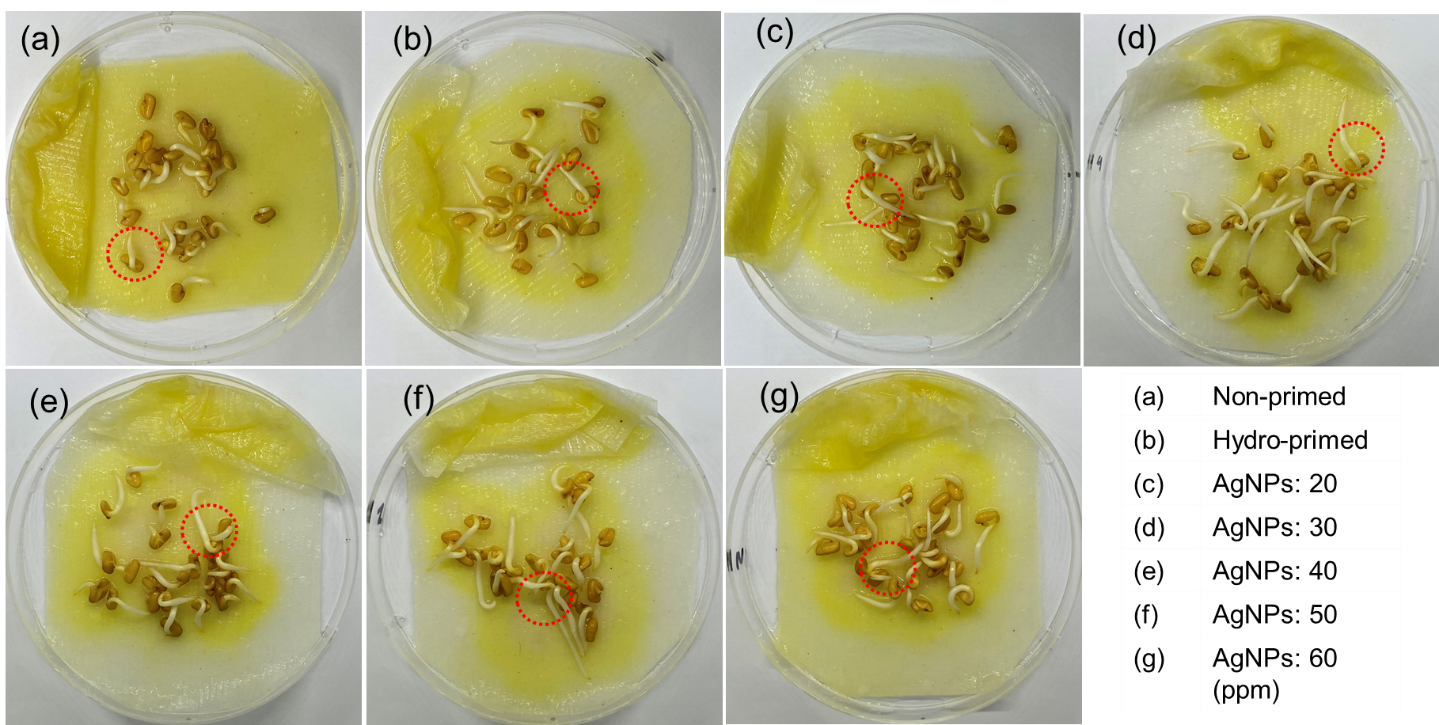


**Fig. S7.** Effect of seed priming with an aqueous dispersion of silver nanoparticles (AgNPs) on the germination of *Trigonella foenum-graecum* seeds, observed at 36 hours post-treatment. The figure illustrates the comparative germination rates between seeds treated with AgNPs and control groups.


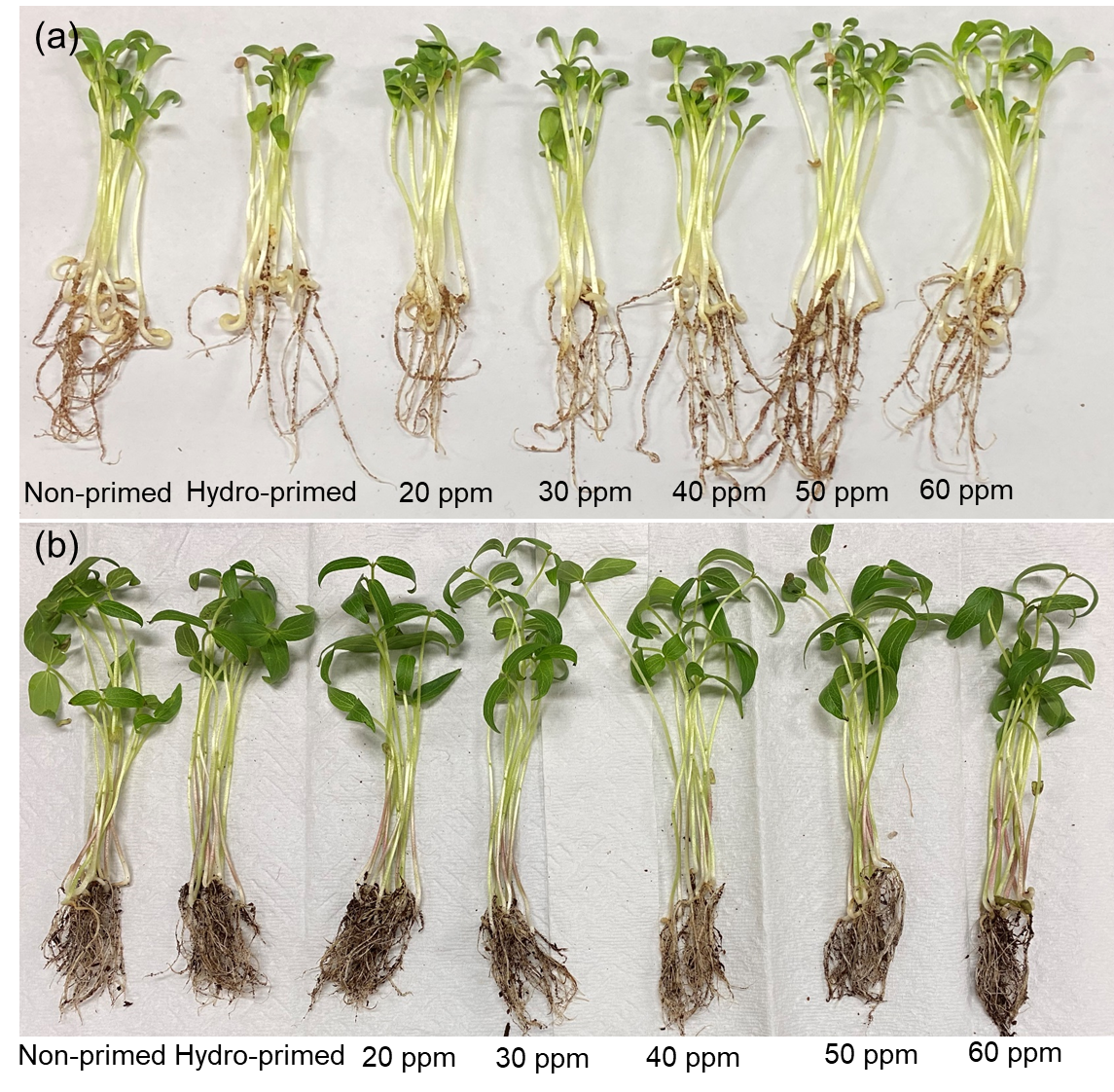


**Fig. S8.** Effect of seed priming with silver nanoparticles (AgNPs) on seedling growth, observed on the 5th day post-germination, for (a) *Vigna radiata* and (b) *Trigonella foenum-graecum*. Seeds primed with AgNPs to control seedlings treated with distilled water, measuring seedling height and root length.
